# Human heat sensation: A randomized crossover trial

**DOI:** 10.1126/sciadv.ado3498

**Published:** 2024-09-04

**Authors:** Stefan Heber, Felix Resch, Cosmin I. Ciotu, Andreas Gleiss, Ulrike M. Heber, Andrea Macher-Beer, Samantha Bhuiyan, Markus Gold-Binder, Renate Kain, Sabine Sator, Michael J. M. Fischer

**Affiliations:** ^1^ Institute of Physiology, Center for Physiology and Pharmacology, Medical University of Vienna, Vienna, Austria.; ^2^ Institute of Clinical Biometrics, Center for Medical Data Science, Medical University of Vienna, Vienna, Austria.; ^3^ Department of Pathology, Medical University of Vienna, Vienna, Austria.; ^4^ Division of Special Anesthesia and Pain Medicine, Department of Anesthesia, Intensive Care and Pain Medicine, Medical University of Vienna, Vienna, Austria.

## Abstract

Sensing of noxious heat has been reported to be mediated by TRPV1, TRPA1, TRPM3, and ANO1 in mice, and this is redundant so that the loss of one receptor is at least partially compensated for by others. We have established an infusion-based human heat pain model. Heat-induced pain probed with antagonists for the four receptors did not match the redundancy found in mice. In healthy participants, only TRPV1 contributes to the detection of noxious heat; none of the other three receptors are involved. TRPV1 inhibition reduced the pain at all noxious temperatures, which can also be seen as an increase in the temperature that causes a particular level of pain. However, even if the TRPV1-dependent shift in heat detection is about 1°C, at the end of the temperature ramp to 52°C, most heat-induced pain remains unexplained. This difference between species reopens the quest for the molecular safety net for the detection of noxious heat in humans.

## Teaser

Human heat nociception relies on TRPV1 but not on TRPA1, TRPM3, or ANO1.

## INTRODUCTION

Avoidance of potentially damaging heat is important but not well understood in humans. There are several heat-sensitive ion channels, whose manipulation led to more or less pronounced phenotypes in animals (*1*–*3*). However, all of the respective knockout animals had, at best, a partial loss in their ability to respond to noxious heat, e.g., considering TRPV1 (*4*) or TRPM3 (*5*) and TRPA1 (*6*). Further attempts have been made to address a potential redundancy in heat pain sensors, but also double knockout animals showed at best minor differences in noxious heat detection, e.g., TRPV1/TRPV2 (*7*), TRPV3/TRPV4 (*8*), as well as TRPV1/TRPM3, TRPV1/TRPA1, and TRPM3/TRPA1 (*9*). In mice, heat sensation was largely absent in triple knockout mice lacking TRPA1, TRPV1, and TRPM3. In animals lacking all three receptors, heat avoidance in hot-plate and tail-flick assays was abolished (*9*). An additional potential heat sensor is Anoctamin 1 (ANO1, also called TMEM16A), which is activated above 44°C in cellular models (*10*). As for the TRP channels discussed above, also ANO1-deficient mice had reduced noxious heat sensitivity but were also still well protected against burn injury.

We addressed by pharmacological means if these results from animal studies translate to humans. The applied concentrations of TRPV1 antagonist BCTC [4-(3-chloro-2-pyridinyl)-*N*-[4-(1,1-dimethylethyl)phenyl]-1-piperazinecarboxamide] and TRPA1 antagonist A-967079 [(1*E*,3*E*)-1-(4-fluorophenyl)-2-methyl-1-pentene-3-one oxime] have previously been validated by inhibition of pain induced by agonists of the respective targets in humans (*11*, *12*). TRPM3 can be inhibited by naringenin [(*S*)-5,7-dihydroxy-2-(4-hydroxyphenyl)chroman-4-one] with a median inhibitory concentration (IC_50_) of 0.5 μM (*13*), and own experiments are in line with the reported results. A concentration of 20 μM naringenin, exceeding the IC_50_ 40-fold, was used to block TRPM3 in humans. There is no systemic toxicity of the locally applied naringenin in the present study as this dose is well exceeded by dietary intake of citrus fruits. On the contrary, there is no dietary TRPM3 blockade as naringenin intake does not lead to pharmacologically relevant local concentrations. Ani9 [2-(4-chloro-2-methylphenoxy)-*N*-[(*E*)-(2-methoxyphenyl)methylideneamino]acetamide] inhibits human ANO1 with an IC_50_ of 77 nM (*14*). It has not been used in humans before. For the approval of using the microdose of 11 μg of Ani9 in human participants, a toxicity study according to the European Medicines Agency International Council for Harmonisation of Technical Requirements for Registration of Pharmaceuticals for Human Use EMA ICH guideline M3 (R2) was conducted. Mice exposed to Ani9 at a 1000-fold higher dose per body weight than for the human trial showed no signs of toxicity 1 and 14 days after exposure compared to controls. As for the other substances, even assuming some local dilution at the injection site, the used concentration of 10 μM in our study should fully inhibit ANO1 by exceeding the IC_50_ 130-fold.

**Fig. 1.**
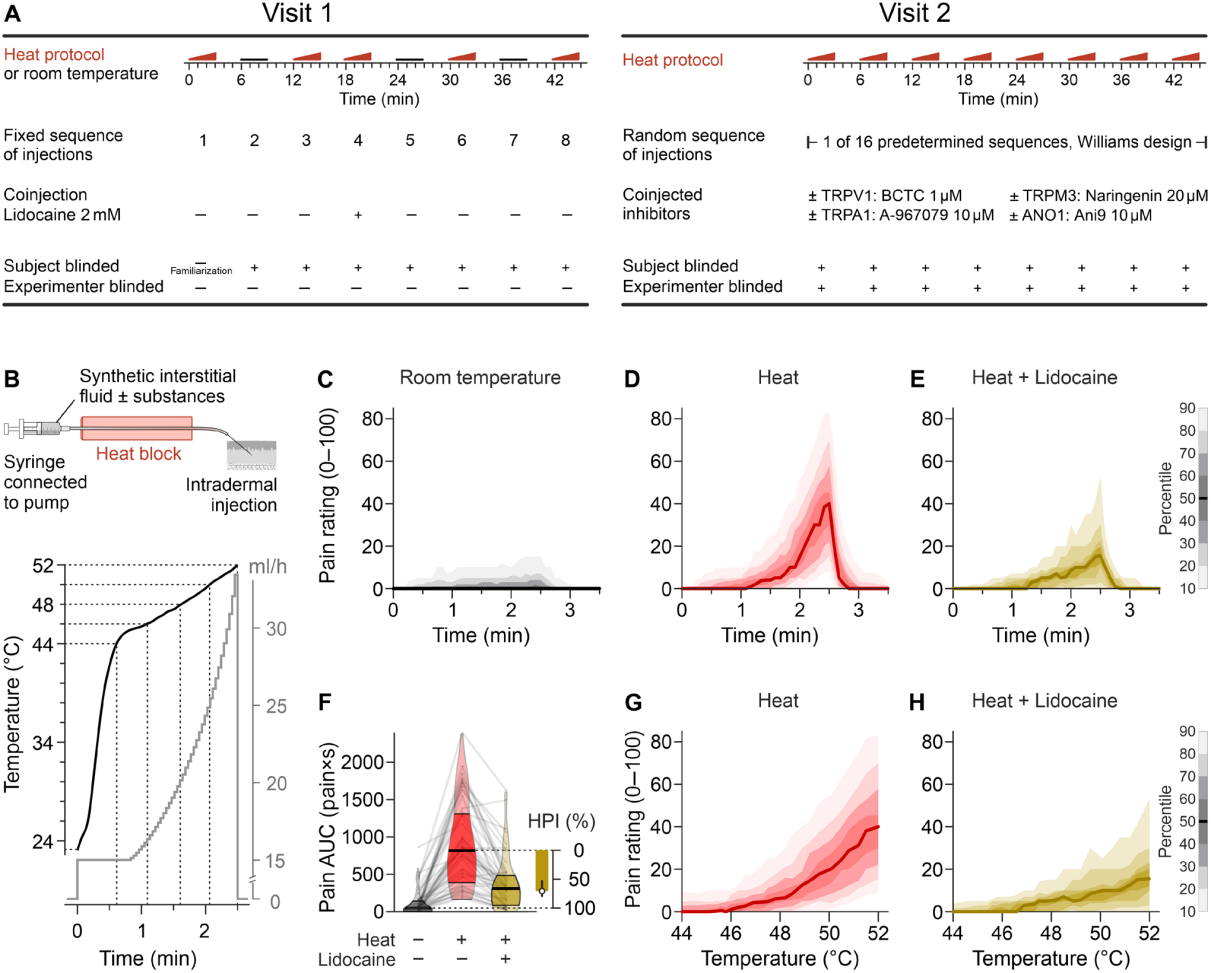
Study design and experimental heat pain model. (**A**) Overview for both study visits. Visit 1 established the infusion heat pain model, including modulation by coinjection of lidocaine. Visit 2 consisted of eight sequential injections, performed double-blinded according to 1 of 16 predetermined sequences. (**B**) From a syringe filled with synthetic interstitial fluid ± test substances, the solution is passed through a heat block with constant temperature and equilibrates to that temperature. The injection rate determines the time for cooling toward room temperature before the fluid reaches the intradermally positioned 27-gauge needle. Nonlinear increase in injection rate (gray) allowed to obtain a largely linear temperature ramp at the outlet in the range of 44° to 52°C. The total injection volume is 812 μl. (**C**) Time-dependent numerical pain rating in single-blinded experiments every 5 s on a 0 to 100 scale. There was minimal reported pain for the three injections at room temperature. (**D**) In contrast, the three injections with increasing temperature generated substantial pain, which rapidly subsided at the end of the 2.5-min injection. (**E**) Lidocaine (2 mM) served as a positive control for pain reduction by a substance added to the injection. The distribution is visualized with the median as a solid line and decreasing gray or color shades for percentiles more distant from the median (in 10% percentile steps, as indicated by the scale bar). (**F**) Pain AUC for injections at room temperature, heated injections, and heated injections with 2 mM lidocaine. Violin plot, indicating the median, interquartile range, and distribution, was overlaid with a spaghetti plot reflecting the 48 individual results. The HPI by lidocaine was calculated; its median (circle) is plotted with the 95% CI. (**G**) Pain induced by heated injections without and (**H**) with lidocaine plotted against the temperature at the needle tip.

With these pharmacological tools at hand, the objective of this study was to test if the redundant function of TRPV1, TRPA1, and TRPM3 observed in mice also applies to humans and if ANO1 is involved in heat perception. According to inclusion and exclusion criteria (table S1), 51 apparently healthy participants were enrolled in the period of 13 June 2022 to 30 November 2022. Of all included participants, 24 male and 24 female participants completed both visits of the study. The first visit validated the pain model regarding its general suitability to detect pharmacological modulation of heat-induced pain. The second visit investigated receptor contribution to heat-induced pain (Fig. 1A).

## RESULTS

### An infusion-based human heat pain model

The heat pain model is based on a short-lasting intradermal injection (2.5 min, 812 μl each) of an increasingly hot interstitial fluid (23° to 52°C; Fig. 1B). Eight subsequent injections were performed at distinct skin sites of the volar forearms. Injection of the room temperature interstitial fluid induced marginal levels of pain (Fig. 1C), which is probably due to local mechanical distension, occuring only in a minority of participants, and the median pain rating is zero throughout the room temperature injections. In contrast, heated injections induced substantial temperature-dependent local pain (Fig. 1D and fig. S1). The reported pain induced by heated injections was assumed to be the sum of pain induced by mechanical distension and pain induced by heat. Thus, heat-induced pain was defined as the difference between pain induced by increasingly hot injections and pain induced by room temperature injections. The effect of test substances was quantified as the percentage of heat-induced pain that could be inhibited [termed heat pain inhibition (HPI)].

As a positive control, HPI was determined for lidocaine, a voltage-gated sodium channel inhibitor (Fig. 1E), as Na_V_1.7 has been shown to be the most important sodium channel target in peripheral unmyelinated sensory nerves (*15*, *16*) and, in particular, for heat perception (*17*). Lidocaine caused an HPI of ~70% (Fig. 1, F to H), irrespective of whether the full temperature range of 23° to 52°C or only the range 50° to 52°C was considered (fig. S2), confirming that the model can be used for a pharmacological approach. The lidocaine concentration of 2 mM was chosen to avoid off-target effects on TRPV1 and TRPA1 observed at 10 mM (*18*), but HPI might have been even greater in case the Na_V_1.7 IC_50_ of 0.5 mM (*19*) would have been exceeded further. In conclusion, visit 1 established an infusion-based human heat pain model, in which microdoses of ion channel inhibitors added to the infusion allow probing the respective targets for their potential involvement in heat perception. As expected, no systemic adverse effects were observed.

### A randomized controlled factorial crossover trial probing TRPV1, TRPA1, TRPM3, and ANO1 as heat sensors

**Fig. 2.**
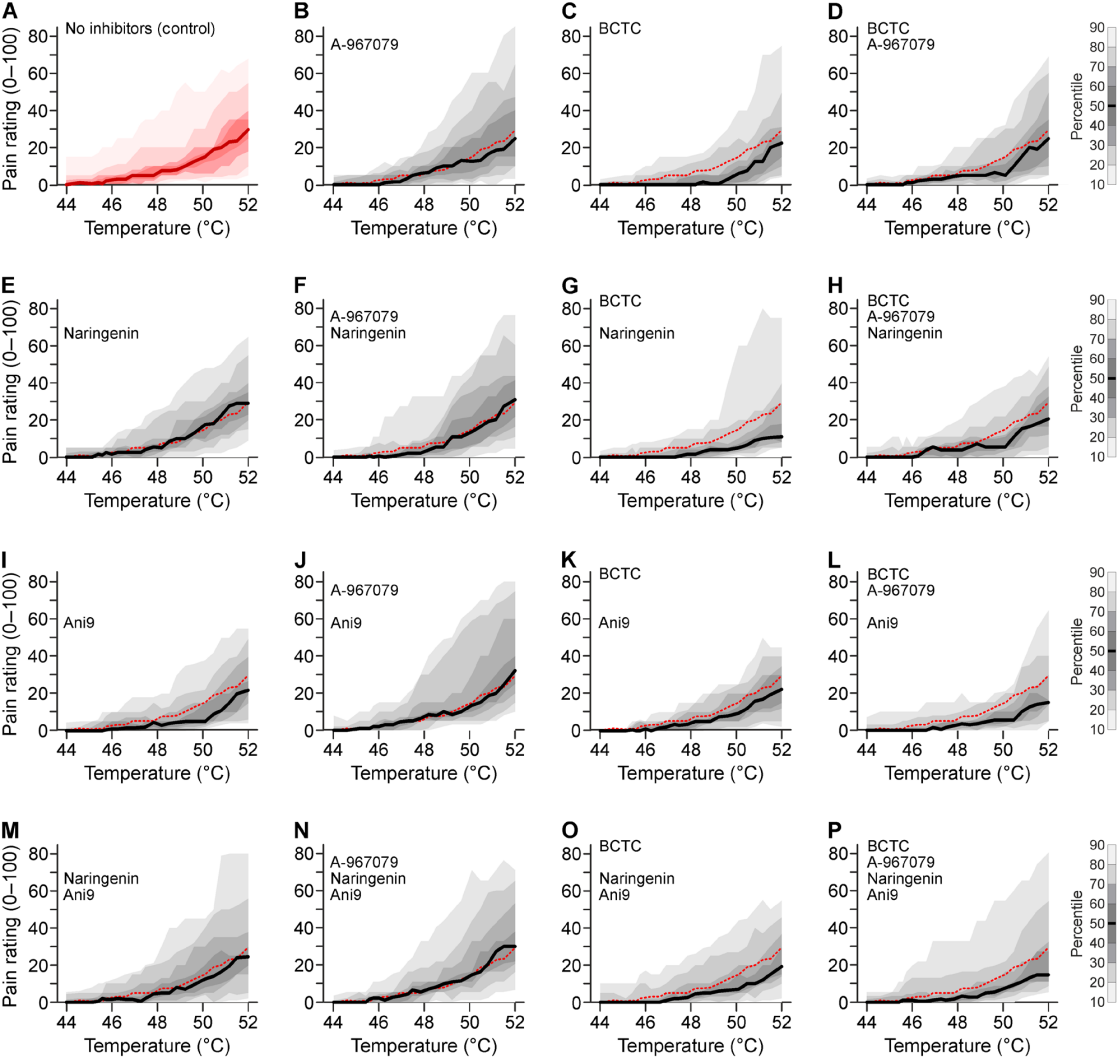
Heat-induced pain ratings in response to pharmacological inhibition of TRPV1, TRPA1, TRPM3, and ANO1. Each panel shows the distribution of pain ratings in the range of 44° to 52°C in visit 2 obtained from 24 participants. (**A**) Pain ratings without antagonists. (**B** to **P**) TRPA1 inhibitor A-967079 (10 μM) is present in panels of columns 2 and 4 and can be compared to the panels to the left of it. TRPV1 inhibitor BCTC (1 μM) is present in columns 3 and 4. Similarly, the TRPM3 inhibitor naringenin (20 μM) is present in rows 2 and 4 and can be compared to the panels above it. The ANO1 inhibitor Ani9 (10 μM) is present in rows 3 and 4. The red dashed line is the median of the control experiment shown in (A). The experimental design derives statistical efficiency from four measurements with and without every antagonist in every participant. Direct statistical pairwise comparisons of panels were formally not justified based on the prespecified design and due to the nonsignificant interaction terms.

The heat pain model was used in a preplanned second visit of the same participants to test if heat pain at the upper end of the temperature range (50° to 52°C) is reduced by simultaneous coapplication of antagonists for TRPV1, TRPA1, TRPM3, and ANO1 (Fig. 2 and fig. S3). The upper end of the temperature ramp was chosen as detection of heat close to a tissue-damaging temperature seemed most relevant for the protective function of pain. Median pain ratings by simultaneous TRPV1, TRPA1, and TRPM3 inhibition (Fig. 2H) as well as by simultaneous TRPV1, TRPA1, TRPM3, and ANO1 inhibition (Fig. 2P and fig. S4) were below controls; however, the difference was considerably less than studies in knockout mice would have suggested (*9*).

Given the described redundancy between the molecular heat sensors, we hypothesized that combinations of inhibitors of TRPV1, TRPA1, TRPM3, and possibly ANO1 would have supra-additive effects. However, based on pain ratings throughout the injection, there was no evidence for this (all interaction terms *P* > 0.16; table S2). The considerable pain levels remaining in the presence of all four antagonists suggest that heat perception in humans, in contrast to mice, relies on further unknown sensors.

### TRPV1 has a role in the perception of heat in humans but TRPA1, TRPM3, and ANO1 do not

**Fig. 3.**
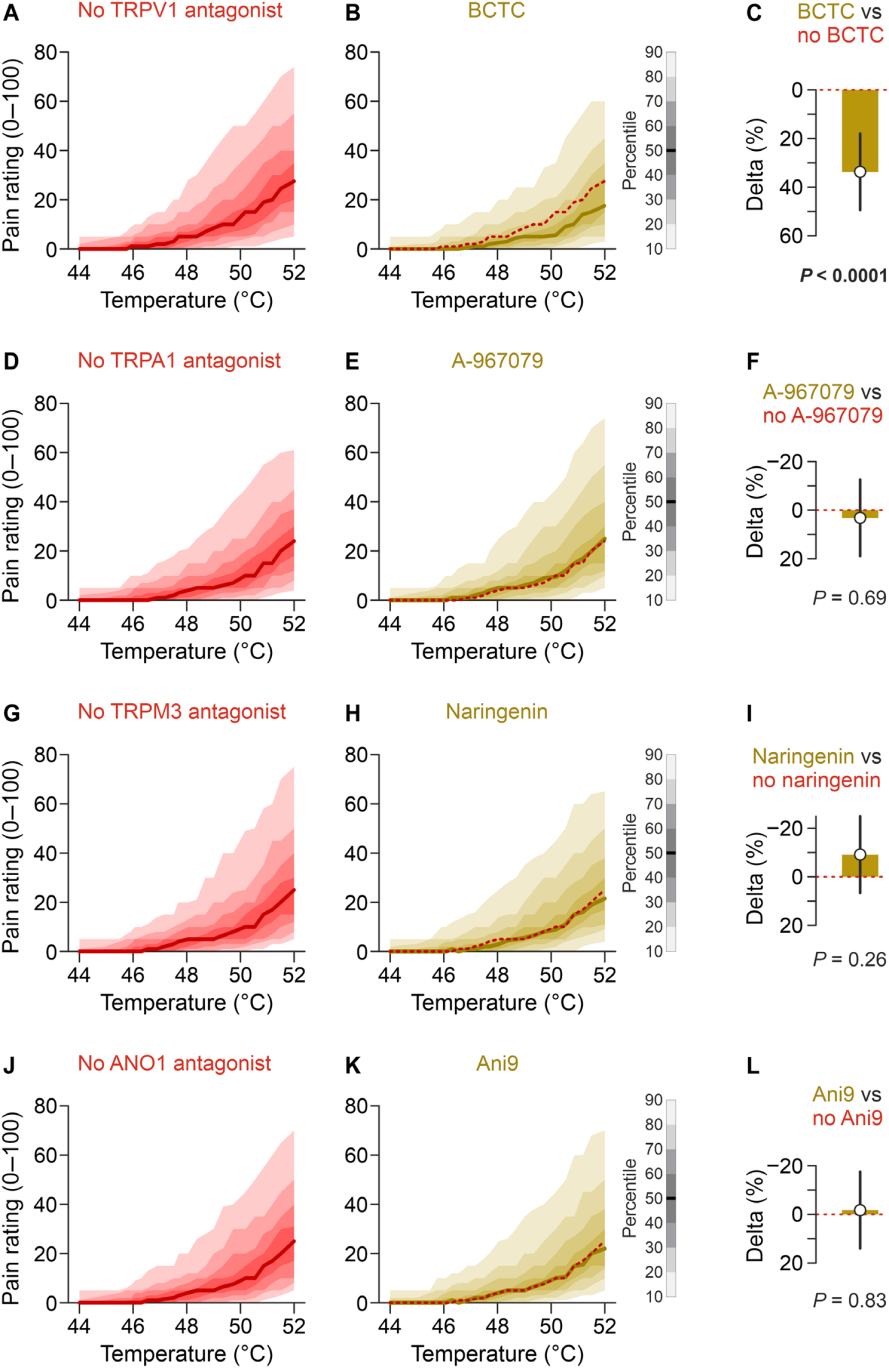
Heat pain is reduced by inhibition of TRPV1 but not TRPA1, TRPM3, and ANO1. Each panel shows the distribution of pain ratings plotted against the temperature. Data are from visit 2 and all 48 participants. The left column shows injections without the respective antagonist but with or without the other antagonists. The middle column consists of all injections including the respective antagonist. (**A**) Pain ratings of all heated injections without and (**B**) with 1 μM BCTC. The red dashed lines indicate the median of all injections without the respective substance. (**C**) HPI due to BCTC over the whole time course (contrast estimate with 95% CI). (**D**) Pain ratings of all injections without and (**E**) with 10 μM A-967079, resulting in (**F**) no relevant HPI. (**G**) Pain ratings of all injections without and (**H**) with 20 μM naringenin, resulting in (**I**) no relevant HPI. (**J**) Pain ratings of all injections without and (**K**) with 10 μM Ani9, resulting in (**L**) no relevant HPI.

The only ion channel that could be confirmed as a heat sensor in humans in this study is TRPV1 (Fig. 3, A and B and figs. S5 and S6). Compared to injections without BCTC, those with BCTC resulted in an HPI that was 33.7% points higher compared to the injections without BCTC [95% confidence interval (CI), 18.0 to 49.5, *P* < 0.0001; Fig. 3C], confirming TRPV1 as a heat sensor in humans. This is consistent with a prior study that has demonstrated the involvement of TRPV1 in human heat sensation by reduced sensitivity after 24 hours high-dose capsaicin desensitization (*20*). Analogous analyses concerning A-967079, naringenin, and Ani9 did not provide evidence that these inhibitors reduced heat pain (Fig. 3, D to L), questioning the role of TRPA1, TRPM3, and ANO1 as heat sensors in humans.

### TRPV1 contribution to human heat perception and what remains for other sensors

**Fig. 4.**
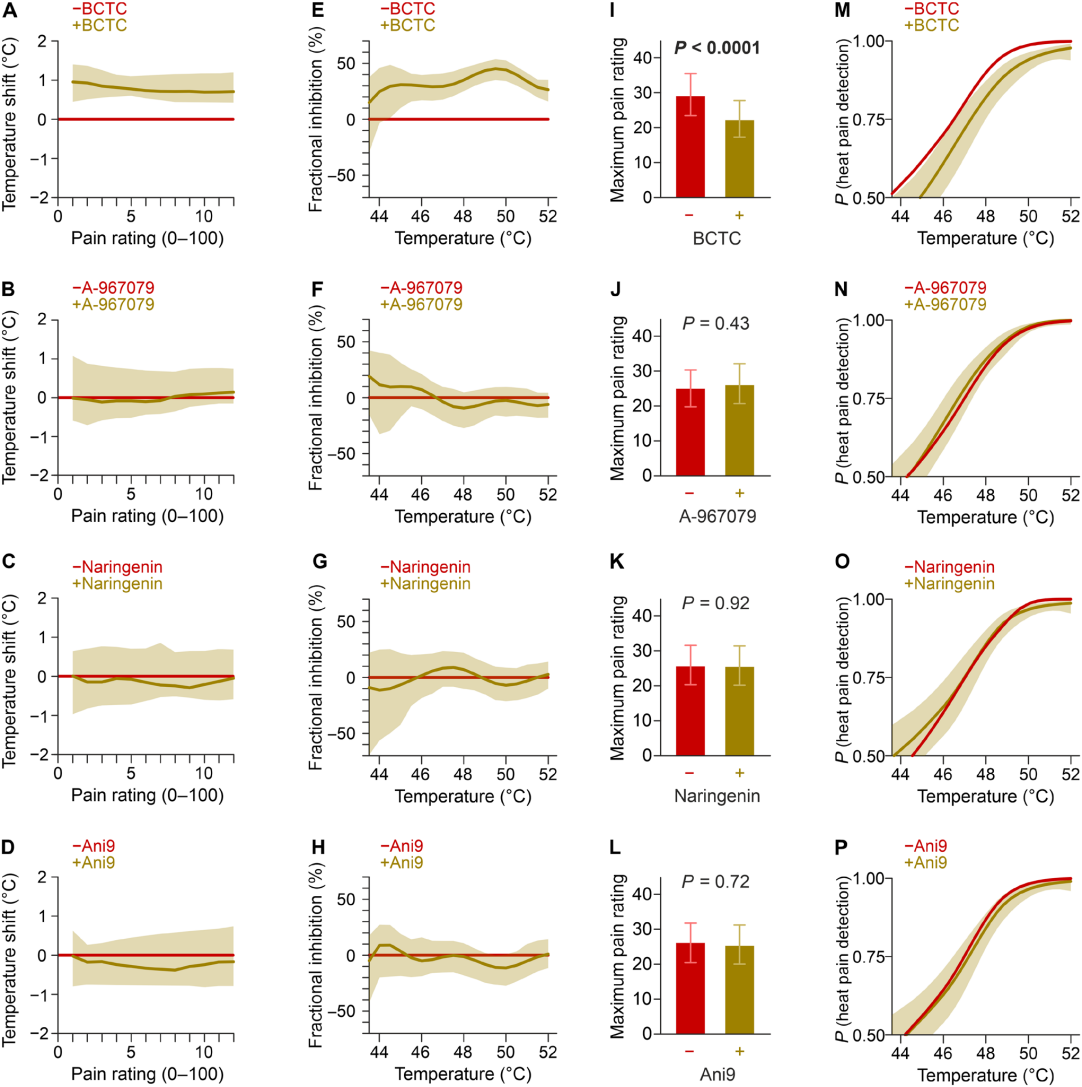
Heat pain threshold and temperature-dependent fractional inhibition. (**A** to **D**) Shift in temperature required to induce the same pain rating. (**E** to **H**) Temperature-dependent fractional inhibition of the heat-induced pain by the antagonist. (**I** to **L**) Maximum pain ratings in injections without the antagonist (red) versus injections with the antagonist (ochre). Data are estimates with 95% CIs. (**M** to **P**) Modeled probability to distinguish the heated injections from the individual three injections at room temperature in the presence and absence of the respective antagonist. Each participant had four injections without the antagonist (red) versus injections with the antagonist (ochre). Data are estimates with 95% CIs.

An antagonist of heat detection should shift the temperature-response curve. This can also be viewed as reduced pain at the same temperature but also as a shift in temperature required to generate the same pain rating. When BCTC was administered, the same pain intensities were reached only at about 1°C more than without (Fig. 4A). This implies that the next-sensitive target has an only slightly higher activation threshold than TRPV1 and not only explains the remaining pain after TRPV1 inhibition but will also contribute to the painfulness of real-life heat stimuli. These results are in line with an increase in the heat pain threshold by 0.6° to 0.8°C reported after oral application of TRPV1 antagonist JNJ-38893777 (*21*), while another study has reported higher increases in heat pain threshold (*22*). It is unclear how much this depends on systemic antagonist exposure and modality-specific antagonism. Nevertheless, in their study, the mean heat threshold was increased from 40.6°C with placebo to 44.8°C; the latter is still recognized as painful and protects the participants from heat-induced damage.

When A-967079, naringenin, or Ani9 were added to the heated fluid, there was no change in the temperature required to induce the same pain rating (Fig. 4, B to D). Next, the fraction contributed to heat perception by the respective receptors was estimated across the temperature range using the pain ratings from visit 2 of injections with and without the respective antagonist. TRPV1 plays a role in heat detection throughout the noxious temperature range with a maximum fraction at around 50°C (Fig. 4E). However, even at this peak, TRPV1 accounts for less than 50% of heat-induced pain. A-967079, naringenin, and Ani9 did not significantly contribute to heat-induced pain at any temperature of the investigated range (Fig. 4, F to H). The presented heat pain model used the full temperature range feasible without tissue damage, which warrants analyzing the maximally occurring heat-induced pain. BCTC in heated infusions reduced the maximally occurring pain rating from 29.2 to 22.2 (*P* < 0.0001; Fig. 4I). As for the other parameters, A-967079, naringenin, and Ani9 did not change the maximum pain rating significantly (Fig. 4, J to L).

Furthermore, it was investigated if a test substance shifted the probability to detect noxious heat. To this end, we first used the pain ratings from room temperature injections (visit 1) in conjunction with the pain ratings from hot injections without BCTC (visit 2). The first seconds of both types of injections barely differ with respect to the temperature of the injected fluid and therefore have similar pain ratings. Thus, the probability that heated injection can be differentiated from a room temperature injection is ~0.5. As the temperature difference between the heated and the room temperature injections increases, there is an increasing probability that pain ratings elicited by the heated injections are higher than those of the room temperature injections, approaching a probability of 1 toward the end of the injection protocol. The presence of BCTC shifted the probability to detect heat-induced pain by about 1°C throughout the observed temperature range (Fig. 4M). A-967079, naringenin, and Ani9 did not shift the probability to detect noxious heat (Fig. 4, N to P).

## DISCUSSION

This investigation was conducted on the skin, and while it is probable that these findings apply to other tissues, this hypothesis remains untested. Contact heat is the established model for assessing human heat pain and is commonly used in clinical settings for quantitative sensory testing (*23*). The developed model builds on the experience gained from pain induced by continuous injection of increasingly acidic fluid (*12*). A key improvement of the injection-based heat pain model is that substances and the thermal stimulus can be delivered to the same site. Application of the substances during the initial nonpainful period will most likely have supplied the site of thermal excitation. The exact site of afferent excitation is unclear for contact heat and heated infusions. It remains an assumption that the sensitivity is similar irrespective of the site, and there is only limited evidence in support (*24*). In contrast, the pilot experiments for the study indicated that it is not trivial to deliver BCTC so that it inhibits contact heat. We have previously shown the effectiveness of BCTC against TRPV1 and A-967079 against TRPA1 in the applied concentration. Evidence is weaker for TRPM3, for which described agonists do not induce pain and therefore do not allow testing of naringenin against an agoinst in humans. Naringenin inhibition was slower to develop compared to other antagonists (*13*), but the lead time of the non-noxious infusion temperatures should cover this period. For ANO1, there is no pharmacological agonist. It is a limitation that it remains unclear if ANO1 activation is sufficient to induce pain in humans and that, without this, there was no model to validate ANO1 inhibition by Ani9 in humans. Furthermore, an interaction of TRPV1 and ANO1 has been described, with ANO1 amplifying the response, being secondarily activated by TRPV1-admitted calcium (*25*, *26*). It should be noted that heat-induced pain in humans did not indicate an interaction between BCTC and Ani9, neither was Ani9 active against heat despite this involving TRPV1 nor was there a sign of Ani9 having a different effect in the presence of BCTC, where there would be nothing to secondarily amplify.

The study tested if the heat perception observed in mice translates to humans. TRPV1 is fully inhibited by BCTC in cellular (*27*) and human experiments (*28*). BCTC can also inhibit TRPM8, but the IC_50_ of 0.8 μM (*29*) is more than 100-fold higher than for TRPV1 (*30*). One might hypothesize that inhibition of TRPM8-related input, interpreted antagonistic to heat-induced pain, might aggravate the latter. However, with local dilution of the 1 μM BCTC injection, minimal TRPM8 inhibition should be assumed and therefore also limited underestimation of the heat-pain reduction through TRPV1. However, human noxious heat perception largely remains in the presence of BCTC, indicating redundancy beyond TRPV1. In contrast to blocking only TRPV1, sensory neurons that express TRPV1 can be defunctionalized by an 8% capsaicin patch. This treatment ablated human heat pain sensation induced by an infrared laser (*20*), indicating that the one or more undiscovered heat sensors are expressed in this neuronal subpopulation. Most hypothesized remaining targets have arguments against them (*31*). One of these targets is TRPM2, which was argued to contribute more in cellular models that investigated higher temperatures (*31*). The lack of suitable pharmacology, conflicting results (*32*, *33*), and the incomplete factorial design discouraged addressing a further target in the present study.

In summary, TRPV1 represents the first line of defense against heat in humans but explains only a limited fraction of the human heat detection beyond the threshold. The remaining targets are unknown, and insights from cellular and animal experiments could inform further investigations in case this puzzle can be solved in such a translational approach. Inhibition of TRPA1, TRPM3, and ANO1 neither shifted the temperature for heat pain detection, nor induced a relevant reduction in pain at any higher temperature, nor reduced the maximal heat-induced pain. The results from combinations of BCTC and the other three substances do not indicate redundancy between the investigated channels. Thus, at least one important additional sensor for heat exists in humans and awaits to be found.

## MATERIALS AND METHODS

### Experimental heat pain model

To expose volunteers to a defined nonhazardous intradermal heat stimulus, a method was established that reproducibly delivers a fluid with a certain temperature into the skin. The buffered interstitial fluid was adapted from a prior description (*34*) and contained 113.8 mM NaCl, 3.5 mM KCl, 1.67 mM Na_2_HPO_4_, 0.7 mM MgSO_2_·7H_2_O, 9.6 mM sodium gluconate, 5.0 mM glucose, 7.6 mM sucrose, 1.5 mM CaCl_2_·2H_2_O, and 22.0 mM histidine chloride, diluted in ultrapure water obtained from a Milli-Q plus system (MQ, Millipore, Burlington, Massachusetts, United States) and 0.4% v/v Tween 80. The solution was adjusted to a pH of 7.4 and sterile filtered using a 0.2-μm filter (Sarstedt Filtropur S). This fluid (not) supplemented with the substances of interest was filled in a 5-ml syringe (B. Braun Injekt Luer Lock Solo), connected to a winged infusion set with a 27-gauge cannula (B. Braun Venofix A, 0.4 × 10 mm, 30 cm).

The tubing was guided over a heat block (CAT Magnetic Hotplate Stirrer M23), fitted with a 20-mm-thick aluminum alloy block (5754 aluminum alloy, Metall Ehrnsberger, Teublitz-Münchshofen, Germany, 135 × 135 mm) on top to increase thermal capacity. This arrangement heats to thermal equilibrium in about 90 min (fig. S8, A and B). For the length of the aluminum, the tubing was embedded in a downward facing cutout (2 × 2 mm) from a custom-cut (175 × 21, 5 × 4, 5 mm) rectangular copper cuboid, improving thermal conduction and limiting heat loss by convection. The length of the heated contact was designed to almost reach thermal equilibrium at the end of the heat block for the used pumping rates (fig. S8C). There was a distance of 80 mm between the end of the heat block and the needle tip. A programmable pump (World Precision Instruments, Sarasota, FL) with a preset time course of a nonlinearly increasing injection rate delivered a total injection volume of 812 μl. Injection speed was constant 15 ml/hour for the first 50 s and then nonlinearly rising to 33.4 ml/hour at the end of the 150-s protocol. This led to a largely linear temperature ramp in the range of 42° to 52°C, sequentially optimized and then 10-fold validated by measurement with a thermocouple at the needle tip and connected to a data logger (UBS-603, Measurement Computing Corporation).

The spatial temperature distribution over time at the skin was measured with a thermal camera (CompactPRO, Seek Thermal, Santa Barbara, CA, United States; fig. S9 and movie S1). The heat protocol was designed to avoid irreversible tissue damage. Such irreversible damage as a function of temperature in the range 44° to 70°C and exposure period was described using porcine and the human skin (*35*). For the heat protocol in this study, the subthreshold “damage” for each second of exposure was summated. The damage at a given temperature was calculated as the fraction of the time to reach the damage threshold at this temperature. For example, at 50°C, the damage threshold is reached after 103.4 s; therefore, 1 s at this temperature accumulates 1.9% toward the damage threshold. The integral over this damage accumulated to 65% of the lower boundary of irreversible damage with a ramp to 52°C. Note that a ramp with 1°C more throughout reaches 108% of the lower boundary of irreversible damage. The experimenter inspected the volunteer’s forearms 5 min after the last injection. No skin burns of grade 1 or above were observed in the participants. Volunteers were also instructed to report immediately in case of any unexpected observations or adverse effects. A condition requiring medical attention has not appeared in any of the participants.

### Human psychophysical experiments

All participants had two study visits, separated by a minimum of 4 days. Both visits had eight injections each and lasted about 1 hour. Each injection started with insertion of a winged infusion set superficially into the cutis; the pain induced by this insertion was noted. The minimum spatial distance between insertion spots was 3 cm. The pump injection protocol started when the insertion-induced pain had fully subsided. Pain was rated every 5 s using a numerical rating scale from 0 to 100 (0 = no pain; 100 = maximal imaginable pain) from the start of the injection until no pain was reported for 30 s. Thereafter, the cannula was removed.

### Study design

The study was a single-group, randomized, placebo-controlled, crossover trial with an incomplete factorial design. The rationale for the crossover design was based on the following assumptions: (i) Because of generally large interindividual differences in pain perception and pain ratings in the population, it was considered advantageous to have heat-induced pain rated in each participant upon addition of as many combinations of inhibitors as possible. (ii) Participants were exposed to such low doses of inhibitors that only local effects at the site of heat exposure but no systemic effects were considered plausible. For this reason, within one visit, multiple short-lasting heat stimuli (± inhibitors) could be tested with regard to pain intensity at intervals of a few minutes at different sites on both forearms. Occurrence of a carryover effect was accounted for by a Williams design as the pain ratings of one injection might influence the subsequent one.

### Visit 1: Familiarization, reference measurements, and positive control

An unblinded control injection of the interstitial fluid with the temperature ramp to 52°C (no. 1) was performed for familiarization with the experimental protocol and the rating scale. Thereafter, the following seven injections with 150-s duration each were performed in a single-blinded manner with a predetermined sequence: three times without heating of the fluid (nos. 2, 5, and 7), resulting in injection of the interstitial fluid of room temperature over the whole injection period, three times with heating of the fluid (nos. 3, 6, and 8), and once with heating of the fluid and addition of 2 mM lidocaine (no. 4). Blinding for the sequence was achieved by covering the line of sight to the heat block, making it impossible for the participant to judge whether the tubing passes the heat block or not. The room temperature and the heated injections serve as an individual reference and allow results of visit 2 to be expressed on a normalized scale (0 = room temperature injection; 1 = increasing temperature injection). This further allows to calculate a fractional inhibition for all injections of visit 2. The lidocaine injection serves as a positive control for a pain-suppressing substance in this infusion-based heat pain model.

### Visit 2: Main experiment

The purpose of visit 2 was to test the predefined hypotheses. Every participant received eight injections with combinations of the four pharmacological antagonists. There are 16 predefined injection sequences (fig. S10). Notably, not all combinations of inhibitors will occur in each sequence as 8 but not 16 subsequent injections appear feasible in one study visit. In this way, the unbiased estimability of the four-way interaction was sacrificed. The design is based on Theorem 19.11 in (*36*). Both halves of the sequences are Williams designs to balance for sequence and first-order carryover effects but inverted regarding whether a substance is used or not. Randomization was performed in blocks. In blocks of 16 volunteers, each volunteer was randomly assigned 1 of the 16 previously generated sequences. In case a volunteer dropped out, the assigned sequence was returned into the pool of available sequences to ensure that the balanced design is achieved only with volunteers who finished the whole protocol. This procedure was repeated until the target sample size was reached. All injections were performed in a double-blinded manner so that neither the participant nor the experimenter knew the respective assigned sequence. M.G.-B. randomly chose 1 of the 16 predefined sequences in closed and identical envelopes, dissolved the respective substances in the interstitial fluid, and provided them to the experimenter. A minimum period of 2 min separated the last nonzero pain rating from the insertion of the needle for the following injection. There were three experimenters, but both visits of every participant were performed by the same experimenter. This study protocol prespecified all aspects provided below and was approved by the local ethics committee (ethics committee of the Medical University of Vienna, vote 1152/2020). The study was registered at www.clinicaltrials.gov (NCT05275751) and was performed in accordance with the Declaration of Helsinki. The study had an incomplete factorial design. The factors were “TRPV1 inhibitor,” “TRPA1 inhibitor,” “TRPM3 inhibitor,” and “ANO1 inhibitor” each with the levels “inhibitor” or “control.” This allowed to estimate the increase in HPI when the antagonists are applied in combination compared to the sum of their isolated effects by using the parameter estimates of the two-way and three-way interactions.

### Outcomes

The primary hypothesis was tested with the outcome variable HPI_(50-52)_, constituting the percentage of pain AUC (area under the curve; unit: pain-seconds) reduction due to combined addition of the four inhibitors in the temperature range of 50° to 52°C. All secondary hypotheses apply to the outcome variable HPI, constituting the percentage of pain AUC reduction over the whole experimental protocol covering the temperature range of 23° to 52°C. Published results suggested that, primarily, the simultaneous inhibition of TRPV1, TRPA1, and TRPM3 would reduce heat pain (*1*) and double combinations including TRPV1 only to a limited extent (TRPV1+TRPA1 and TRPV1+TRPM3). In the framework of factorial designs, this would correspond to a three-way and two two-way interactions. As reduction in heat pain by any double combination of inhibitors (corresponding to two-way interactions) or even by an inhibitor alone (corresponding to main effects) was considered relevant as well, the secondary hypotheses covered all two-way and three-way interactions as well as main effects. The estimation of the four-way interaction, meaning that heat pain is reduced only if all four receptors of interest are inhibited, was omitted for the following reasons: (i) The number of injections that can reasonably be done within a volunteer’s visit is limited, (ii) the four-way interaction needs the highest sample size for adequate statistical power, and (iii) the four-way interaction can be considered the least biologically plausible interaction.

### Recruitment, participants, and setting

Participants were recruited using a notice distributed at the Medical University of Vienna. Participants between 18 and 70 years of age and full legal capacity were recruited. To ensure that each sex was equally represented in the study population, only participants of one sex were enrolled after the number of participants of the other sex reached half of the calculated sample size.

Exclusion criteria were participation in another study within the past 4 weeks, recent or ongoing medication intake (except for contraceptive drugs), current breastfeeding or pregnancy (test-based exclusion was done in all female participants prior to visit 2), drug abuse, fever, known allergic diseases (in particular asthmatic disorders and/or allergic skin diseases), history of allergic reactions to citrus fruits, sensory deficits, skin disease or hematoma of unknown origin in physical examination of the test site, and a body temperature above 38°C or symptoms of a respiratory tract infection (the latter two as COVID-19–related criteria). Experiments were carried out in the general hospital of the Medical University of Vienna in a room without physical or acoustic disturbance.

Following comprehensive instruction regarding the nature, significance, impact, and risks of this study, participants provided written consent to participate in the study. Furthermore, participants were informed of the possibility to withdraw their consent for any reason at any time. In addition to the instructions given by the experimenter, participants also received an information sheet written in layman’s terms, explaining the nature and purpose of the study and its progress.

### Applied substances, dosage, and administration

All substances were obtained from Sigma-Aldrich; the purity of all substances was specified as at least 98% based on high-performance liquid chromatography (HPLC). It should be considered that a previous study found differences between cellular and apparent IC_50_ values in psychophysical experiments (*11*), and the assumption of an about 10-fold lower effective concentration due to dilution and redistribution served well to choose the concentrations. A-967079 was used in two previous trials approved by the ethics commission (EK nos. 1799/2017 and 1969/2018) and respective publications (*11*, *12*) at a concentration of 10 μM. BCTC was also used in prior acidosis-induced pain studies (*12*, *24*) at a concentration of 1 μM. Naringenin is a bioactive flavonoid found in citrus fruits. At higher concentrations exceeding the ones used in this study, it is investigated for a variety of therapeutic uses (*37*), including antimicrobial, antioxidant, anti-inflammatory, and anti-allergic action. Despite the low oral bioavailability of naringenin, intake of citrus fruits generates systemic exposure (*38*). Grapefruit juice contains naringenin (4 to 31 mg/liter) for instance (*39*). Because of widespread exposure, lack of adverse effects can be assumed, at least for participants without known allergy to citrus fruits. Allergies toward citrus fruits are reportedly rare in central Europe, and the three main orange allergens Cit s 1 to 3 do not include naringenin. Nevertheless, known allergy to citrus fruits was added as an exclusion criterion. Naringenin has a published IC_50_ for TRPM3 of 0.5 μM (*13*) and has been validated on TRPM3 expressed in human embryonic kidney (HEK) 293T cells. On the basis of these data, a concentration of 20 μM was used.

Ani9 is an inhibitor of ANO1 with an IC_50_ of <0.1 μM. As this substance has not been used in humans until now, we performed a toxicity study in mice according to the EMA ICH guideline M3 (R2) for microdosing trials. The toxicity study was applied for and approved by the Austrian Federal Ministry of Education, Science and Research (2020-0.648.592). C57BL/6 mice, 50% females and 50% males, were 6 to 7 weeks of age. Using a concentration of 10 μM, the study dose of Ani9 equals 154 ng/kg for a participant with a 70-kg body weight. A dose of 154 μg/kg was injected in 30 mice, corresponding to a 100-fold relative dose, assuming an allometric scaling factor of 10 from humans to mice. For a mouse of 20-g body weight, a volume of 125 μl was subcutaneously injected on the back of the neck. Twenty animals were assessed 24 hours after injection of Ani9, whereas another 20 animals served as controls being injected with a control solution. Ten further animals were assessed 2 weeks after the injection of Ani9. In accordance with the guideline, this group did not have a control group. For the analysis of microscopic and macroscopic pathological findings, the actual descriptive findings were first simplified to a dichotomous target parameter. All “normal” findings were taken as such; all others were simplified to the category “not normal.” The proportion of non-normal findings was compared between the three groups using the Fisher’s exact test. For paired organs, a generalized mixed linear model with the binary target parameter mentioned above was initially considered to account for the dependence of the findings of a left and right organ within an animal with a random factor “animal.” However, initial analyses did not converge, most likely due to the rare occurrence of the not normal category. Therefore, the results of paired organs were combined. The organ was only classified as normal if both sides were classified as normal; otherwise, it was classified as not normal for the Fisher’s exact test.

The metric variables concerning clinical chemistry and blood count were compared between the three groups by the Kruskal-Wallis test; correction for multiple comparisons within each outcome parameter was done by the Dunn’s method. Experiments were conducted in the Center for Biomedical Research of the Medical University of Vienna being certified for good laboratory practice. Analyses included hematology, clinical chemistry, autopsy, and histopathology and were based on Hayes’ Principles and Methods of Toxicology. Hematological analysis included erythrocyte count, hemoglobin, hematocrit, mean corpuscular haemoglobin, mean corpuscular haemoglobin, mean corpuscular haemoglobin concentration, red cell distribution width, reticulocyte count, leukocyte count, differential blood count, platelet count, and mean platelet volume. After 24 hours, no changes were observed in Ani9-treated animals compared to controls. However, there were changes in the red blood cell indices 14 days after Ani9 injection (fig. S11). Therefore, in an independent experiment, 10 mice each were compared after 14 days of injection with Ani9 or control. For each animal, a baseline blood count and another blood sample after 14 days were obtained. These experiments were analyzed using analyses of covariance. The values collected immediately before injection were used as covariates, Ani9 or control as a binary factor and the values 14 days after injection as the dependent variable. Addition of the 14-day controls again showed a time-dependent change in some of the parameters, but none of those were different between controls and Ani9-treated animals (fig. S12). Clinical chemistry analysis included glucose, blood urea nitrogen, creatinine, total protein, albumin, globulins, inorganic phosphate, calcium, sodium, potassium, chloride, total bilirubin, cholesterol, triglycerides, alkaline phosphatase, aspartate aminotransferase, alanine aminotransferase, γ-glutamyltransferase, and ornithine transcarbamylase and did not yield relevant changes of Ani9-treated animals compared to controls either (fig. S13). Autopsy and histopathology were performed by trained pathologists and included removal, weighing, and macroscopic analysis, followed by histopathological evaluation of the injection site, the heart, the lung, the thyroid gland, the submandibular and parotid glands, the larynx, the esophagus, the abdominal cavity, the stomach, the pancreas, the gallbladder, the cecum, the lymph nodes, the spleen, the thymus, the eyeball, the brain, the adrenal gland, the kidney, the urinary bladder, the uterus and uterine tubes, the ovaries, the testis, the epididymis, and the prostate. On macroscopic and histopathological levels, no pathology caused by Ani9 could be detected in any of the organs (fig. S14). In summary, the mouse microdosing study had not yielded results that opposed the use of Ani9 in the present human study.

Particularly considering the TRP channels, there are agonists and antagonists affecting more than one target. To this end, the receptor specificity of the used substances and concentrations was investigated in HEK293T cells expressing the respective human TRP channels. (fig. S15).

HEK293T cells grown in Dulbecco’s minimum essential medium (D5648, Sigma-Aldrich), supplemented with penicillin, streptomycin, and l-glutamine (1% each, all from Lonza, Basel, Switzerland), were transfected using a jetPEI transfection reagent (Polyplus, Illkirch, France). The cells were then spread on poly-d-lysine–coated black 96-well plates (~30,000 cells per well) and incubated overnight at 37°C and 5% CO_2_. The microfluorimetry of cytosolic calcium levels was performed with calcium 6 (Calcium 6 kit, Molecular Devices, San Jose, CA) at 37°C. A pipetting fluorescence plate reader (FlexStation 3, Molecular Devices, San Jose, CA) was used to excite every 2 s at 485 nm, and the AUC of fluorescence emission served as an index of intracellular calcium responses. Human TRPA1 activation by allyl isothiocyanate was fully inhibited by A-967079, also when all four antagonists were present but not by the other three antagonists. Human TRPV1 activation by capsaicin was fully inhibited by BCTC, also when all four antagonists were present but not by the other three antagonists. Human TRPM3 was activated by the combination of CIM0216 and pregnenolone sulfate, each 2.1 μM, as this generates a supra-additive effect. However, only one of the two TRPM3 activations can be inhibited by the antagonist (*40*), and an incomplete reduction by naringenin was observed as expected. As before, the level of inhibition was not different in the presence of all four antagonists, and the other three antagonists combined did not reduce TRPM3 activation. There is no agonist for ANO1, prohibiting assessment as for the TRP channels even if there would be a dye with sufficient sensitivity to detect induced changes in the chloride concentration. However, there is no evidence that the specific TRP channel antagonists act on the unrelated group of calcium-activated chloride channels to which ANO1 belongs.

Considering potential adsorption by the polyethylene tubing and/or lack of heat stability of substances, solutions that would have been injected from a regular heated protocol were sampled in five 30-s fractions and measured by HPLC (fig. S16). Acetonitrile HPLC grade was obtained from VWR Chemicals (Fountenay-sous-Bois, France). Substances were dissolved in deionized water and quantified using RP-C18 columns (150 × 3.0 mm, 2.5 μM; Kinetex, Phenomenex) in a Dionex Ultimate 3000 UHPLC (Thermo Fisher Scientific, Waltham, MA). The mobile phase was a gradient from solvent A (water with 0.05% trifluoroacetic acid) and solvent B (10% water and 90% acetonitrile with 0.05% trifluoroacetic acid) with a flow rate of 0.3 ml/min. The gradient started at 95% A/5% B and was linearly raised to 85% B over a period of 40 min. Absorbance was monitored at 280 nm. To obtain the target concentrations in the actually injected solutions, substance loss was compensated by adapting the amount of substances added to the initially prepared solutions. Compensation factors for the respective substances were as follows: lidocaine, 1.20; BCTC, 1.27; A-967079, 1.28; naringenin, 1.00; and Ani9, 1.19, which is in line with expectations from the respective xLogP3 values (*41*).

All four antagonists are small molecules without a highly reactive chemical structure. Nevertheless, temperature stability and lack of chemical interaction of the four substances were investigated by HPLC. The four antagonists were mixed and heated to 72°C for 10 min. The HPLC of the heated mixture showed a similar signal for each substance as the separate run of each substance alone, with and without prior heat exposure. Heat stability of the antagonist combination was further tested by heating for 3 min to variable temperatures. Also in these experiments, exceeding the thermal exposures of the heat pain model, more than 97% of all substances were recovered after exposure to 72°C (fig. S17).

### Statistical methodology and analysis

Outcome variables were based on the pain ratings acquired during both visits. After data acquisition, the following steps were performed for each participant separately to calculate outcome variables. Pain ratings of the three heated injections of visit 1 were averaged for each 5-s time point. Accordingly, ratings of the three unheated injections of visit 1 were averaged. As an integral measure of pain, for the resulting reference curves, pain AUC values were calculated using the trapezoidal rule. Next, the pain AUC obtained from the room temperature injection (considered as mechanically induced pain) was subtracted from the one obtained from the heated injection, resulting in an area reflecting exclusively heat-induced pain. The HPI was calculated for all substances and/or combinations of substances, serving as a primary outcome variable. HPI was defined as dividing the difference between averaged heated injections (visit 1) and the respective heated injection (visit 2) by the difference between averaged heated and unheated injections (visit 1), multiplied by 100 to provide a percent measure. The percentages reflecting HPI in the range of 50° to 52°C represent the primary variable HPI_(50-52)_, used to test the primary hypothesis. The fractional inhibition over the whole temperature range is calculated in analogy and serves as the secondary outcome variable HPI, used to test the secondary hypotheses.

### Statistical model for primary analyses

An initial data analysis identified a few extreme outliers at the lower tail of the distribution of both outcomes, HPI_(50-52)_ and HPI. Consequently, both outcomes were winsorized at their fifth percentile across all observations before used as dependent variables in the models described below.

In the prespecified statistical approach, a linear mixed model was applied with the primary outcome variable HPI_(50-52)_ of injections of visit 2 (all inhibitor combinations) as a dependent variable. The predictors were the binary within-participant factors “TRPV1 antagonist,” “TRPA1 antagonist,” “TRPM3 antagonist,” and “ANO1 antagonist” each with the levels inhibitor (substance used) and control (substance not used). To account for the potential correlation between pain AUC values from the same volunteer, a random participant factor with an AR(1) structured covariance pattern (a first-order autoregressive structure with homogenous variances) or a Toeplitz structure or compound symmetry was prespecified to be chosen based on the lowest Akaike information criterion. Considering the crossover design, the position in the injection order (period) was included in the model as a categorical factor with eight levels. In a prior study (*11*), insertion pain was a minor predictor of the subsequent pain induced by the experimental protocol; this was confirmed in the present study (fig. S18). As prespecified, to adjust for differences in insertion-induced pain between injection spots, the respective values were included as a covariate after log-transformation to counterbalance its skewed distribution. To test the primary hypothesis, the factorial model with all possible interactions except the four-way interaction was used, with a contrast comparing the HPI_(50-52)_ values between the injections without any substance and the one with the quadruple combination. As a positive control, the primary outcome variable HPI_(50-52)_ of the lidocaine-containing heated injections was tested against 0 with a one-sample *t* test. To test the secondary hypotheses, the outcome variable HPI was used. Nonsignificant interaction terms were removed until only significant interaction terms remained or only main effects were left. Nonsignificant three-way interaction terms were omitted from the model first. Following this, all nonsignificant two-way interactions were omitted, beginning with the one with the highest *P* value, unless they are part of significant three-way interactions. Least-squares means are reported from the final model with 95% CIs. Contrast estimates were plotted with their respective 95% CIs. Significant interactions were sliced using appropriate contrasts. In an exploratory approach, a “fractional inhibition” by an antagonist quantified the respective receptor contribution at a particular temperature.

Conditional residuals of each linear mixed model were inspected for gross deviations from normal distribution and overly influential observations; none of which were observed. All reported *P* values are the results of two-sided tests. *P* values of ≤0.05 were considered statistically significant. No correction for multiple testing was performed due to the prespecification of a single primary contrast. The statistical analysis was carried out using IBM SPSS statistics 28 and SAS 9.4. Graphs were generated using GraphPad Prism 9 or R with the ggplot2 data visualization package.

### Sample size calculation and internal pilot

Sample size was determined according to the first secondary hypothesis so that the detection of a relevant three-way interaction of 25% points HPI is possible with a power of at least 90%. This involves a sufficient sample size for detecting a relevant effect of 50% points HPI_(50-52)_ regarding the primary hypothesis. Assuming two-way interactions to be scientifically equally important, an effect of 25% points HPI would be relevant. Similarly, an effect of 25% points HPI would be relevant for possible main effects.

An adequate sample size was prespecified to be determined by simulations based on an internal pilot with *N* = 16, without evaluation of the primary and secondary hypotheses. Simulations were conducted after completion of the first block of 16 participants and exclusively based on the residual variance-covariance structure. As the estimated effect size was not taken into account, type 1 error inflation seems negligible. Simulations were performed using SAS 9.4 with a model identical to the analysis model described above. Simulations used the a priori defined minimally relevant effect size of 25% points HPI for a three-way interaction with variance and correlations estimated from the internal pilot data. Simulations were designed to show the necessary sample size to detect the relevant effect with a power of 90%, accepting the two-sided probability of a type I error of 5%.

There was a prespecified decision tree after the internal pilot, approved by the ethics committee. The flow chart includes the results of the simulation-based sample size calculation (fig. S19). On the basis of the simulation, more than 32 volunteers would be necessary to detect a relevant effect size of a three-way (power of 17%) and a two-way interaction (power of 51%), indicating that the observed intra-individual variance was larger than anticipated and therefore the study underpowered to detect these interactions. For the main effects, *n* = 16 was estimated to provide a power of 81% and *n* = 32 for a power of 97%. For the primary hypothesis, which was not considered for sample size calculation, the simulation-based estimated power for 32 volunteers was 68.7%. This was considered insufficient, and due to the Williams design, a study extension by multiple of 16 was considered. On the basis of residual variance, the simulation for 48 participants calculated a power of 85.7% for the primary hypothesis. Therefore, the study protocol was amended to complete 48 participants in total, which was approved by the ethics committee. Data were analyzed when 48 participants had completed the protocol.

A dropout rate below 10% was assumed, resulting in an approval to include 48 +4 volunteers to enable per protocol analysis of 48 participants. A total of 51 volunteers came to the first visit; two decided against participating in the second visit due to time restrictions, and for one further participant, the second visit could not be completed due to loss of a (blinded) syringe, resulting in 24 female and 24 males who completed both study visits. The age range of participants was 20 to 32 years (female median, 23.8; interquartile range, 21.9 to 25.6; male median, 23.1; interquartile range, 21.9 to 25.7).

### HPI by lidocaine

The median and its CI were calculated for the HPI by lidocaine.

### Fractional inhibition over the temperature range by each substance

For use as a dependent variable, raw pain ratings of visit 2 were log-transformed after adding 1 to incorporate zero ratings. The linear mixed model includes indicators for the four antagonists as well as injection order (period) and log-transformed insertion pain as fixed factors. Furthermore, terms representing restricted cubic splines of temperature (only values of 43.5° and higher were included), with knots set at the three quartiles, and their interaction with the considered antagonist were included. Besides a random intercept for each participant, a random intercept and a linear, quadratic, and cubic temperature term were included within each participant-period combination. Least-squares means were estimated at various temperatures to compute fractional inhibition. Bias-corrected and accelerated (BCa) bootstrap 95% CIs for fractional inhibition were estimated from 250 randomly drawn participant-clustered bootstrap samples to correctly account for the shifted log-transformation of the dependent variable.

### Effects of substances on maximum heat pain

A linear mixed model was used with square-root transformed individual maxima as a dependent variable and the four antagonists as well as injection order and log-transformed insertion pain as fixed factors. The correlation of repeated measurements was modeled using an AR(1) structure.

### Probability to detect heat-induced pain

The probabilities to rate pain due to the increasingly hot injections higher than the room temperature injections as a function of temperature were estimated as follows: First, an extended dataset was built that contained, at each temperature, an indicator if the pain rating was higher with or without the considered antagonist. Data lines for unambiguous cases received a weight equal to one. If the two ratings coincided, then the data line was included twice, with the indicator set to one and set to zero, but both time with weight set to one-half. This binary indicator was then used as a dependent variable in a generalized linear mixed model with quadratic spline terms for temperature and their interaction with the considered antagonist. The intercept and the main effect of the antagonist were suppressed to force a probability of 0.5 with and without the antagonist at the rescaled temperature of zero, which corresponds to the minimum temperature of 23°C. A random participant effect was added allowing for different estimates with and without the considered antagonist. The predictions with and without the antagonist were estimated from this model. BCa bootstrap 95% CIs for fractional inhibition were estimated from 250 randomly drawn participant-clustered bootstrap samples.

### Shift of temperature necessary to elicit a certain pain rating

To estimate the shift of temperature necessary to elicit a certain pain rating induced by substances, the following calculations were performed for each participant individually. First, the following dose-response curve was fitted to pain ratings versus temperature. *Y* = Bottom + *X*^Hillslope^ × (Top − Bottom)/(*X*^Hillslope^ + EC_50_^Hillslope^), where *Y* = pain (0 to 100), *X* = temperature, and Top and Bottom are the asymptotes. As each injection started with a pain rating of 0 at room temperature, the parameter “Bottom” was constrained to a pain rating of 0. Although the temperature ramp was designed to avoid tissue damage, we assume that heat-induced pain can, in principle, reach a maximal imaginable pain. Thus, the parameter “Top” was constrained to a pain rating of 100, resulting in the two parameters “Hillslope” and “EC_50_” to be estimated: *Y* = *X*^Hillslope^ × 100/(*X*^Hillslope^ + EC_50_^Hillslope^). For each inhibitor, data of the four injections without and the four injections with the inhibitor were used. This implicitly assumes that the absence of interactions between ion channels found in the main analysis also holds true for each temperature. Curve fitting resulted in two dose-response functions for each of the four inhibitors in each volunteer. For each of these, the temperature at a pain rating of 1 to 12 was interpolated. This provided a temperature difference at a given pain level for each inhibitor for each volunteer. The median and 95% CI of this temperature difference were plotted for each pain rating in Fig. 4 (A to D).

During their participation in this trial, all volunteers were insured by the “Rahmenversicherung MedUni Wien.” Digital data processing occurred in an anonymized form by assigning sequential numbers to each participant and thus conforms with the European law on data protection (Datenschutz-Grundverordnung).

The study followed the International Council for Harmonisation of Technical Requirements for Registration of Pharmaceuticals for Human Use good clinical practice guidelines and the regulatory requirements and therefore the EU Directive embedded in the Austrian Drug Act. The study provides all data requested by the Consolidated Standards of Reporting Trials for crossover trials.

## Supplementary Materials

### The PDF file includes:

Figs. S1 to S19 Tables S1 and S2 Legend for movie S1 References
