# Supplementary material for: Human heat sensation: A randomized crossover trial
Source: Sci Adv. 2024 Sep 4;10(36):eado3498. doi: 10.1126/sciadv.ado3498 (PMC11373589; doi:10.1126/sciadv.ado3498)
Supplement: Supplementary file 1 — Figs. S1 to S19 Tables S1 and S2 Legend for movie S1 References [file sciadv.ado3498_sm.pdf]

Science Advances
AAAS

Supplementary Materials for

Human heat sensation: A randomized crossover trial

Stefan Heber *et al.*

Corresponding author: Michael J. M. Fischer, michael.jm.fischer@meduniwien.ac.at

Sci. Adv. **10**, eado3498 (2024)
DOI: 10.1126/sciadv.ado3498

The PDF file includes:

Figs. S1 to S19
Tables S1 and S2
Legend for movie S1
References

Other Supplementary Material for this manuscript includes the following:

Movie S1

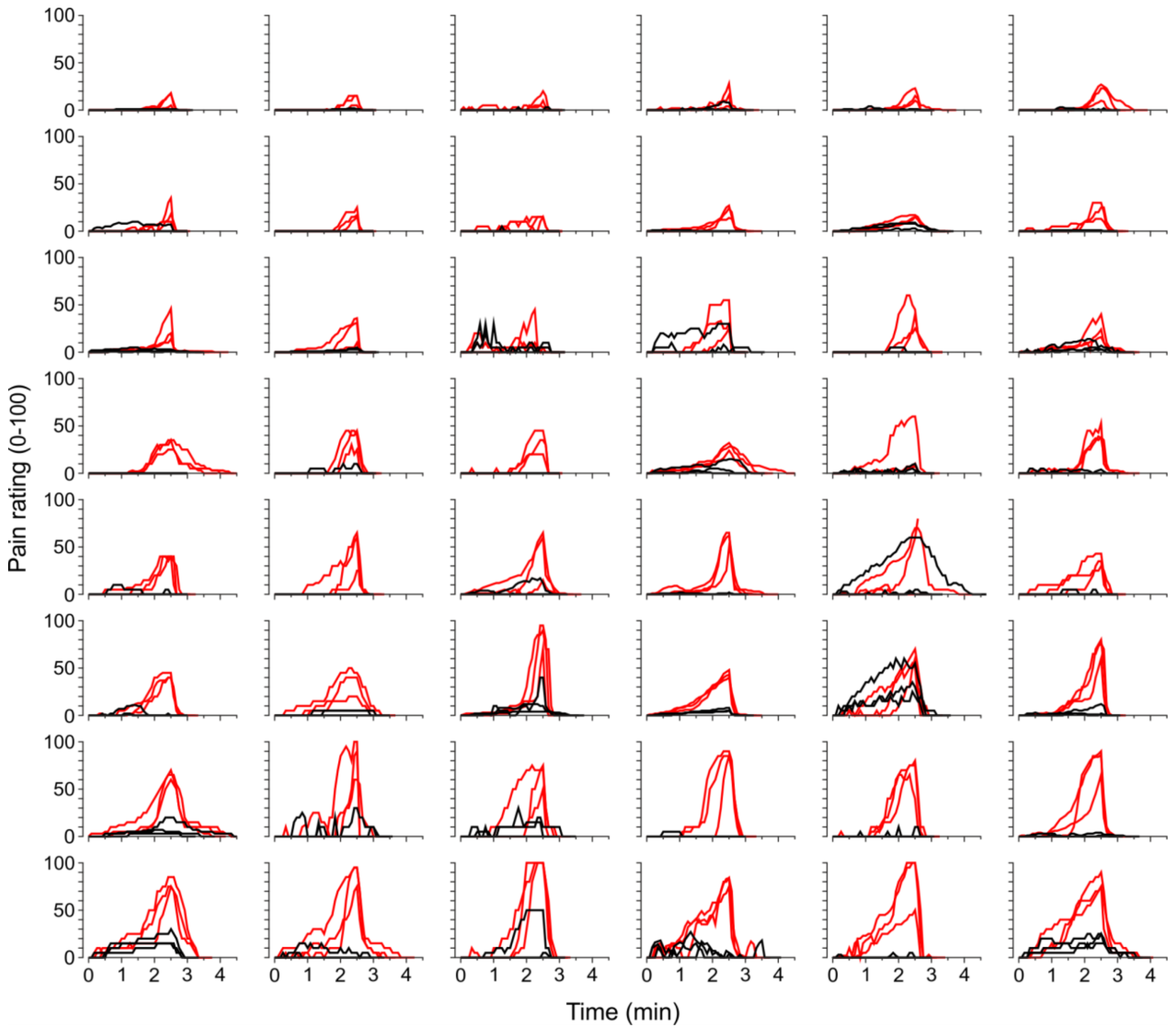

Fig. S1. Intraindividual reproducibility and overall variability. Every panel reflects one of the 48 subjects. Each panel holds the pain ratings for three injections with room temperature (black) and three heated (red) injections of visit 1 for all subjects. The panels are sorted from the smallest to largest mean heat response. The pain AUCs of the three heated injections had 29% deviation (median, interquartile range 20–46%) from the intraindividual mean of the pain AUCs. For the heated injections without lidocaine, females had median AUC pain rating of 983, males of 1608 ($p = 0.027$, U-test). However, this should not be interpreted, as 32 experiments were performed by a male experimenter, and 16 by a female experimenter *(42, 43)*, and the trial was not designed to detect sex differences.

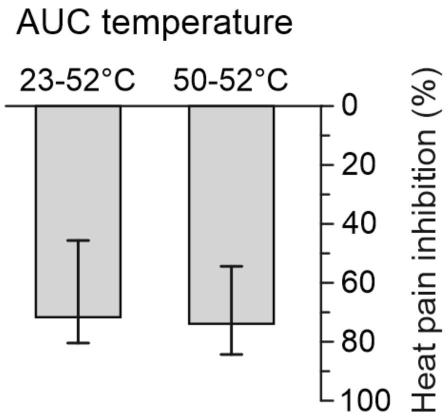

Fig. S2. Comparison of heat pain inhibition based on different temperature ranges. Heat pain inhibition by lidocaine 2 mM was calculated over the full temperature range (23–52°C) or for the range 50–52°C (~last 25 s). Note there is about 70% inhibition with both measures. Error bars are 95% confidence intervals of the median.

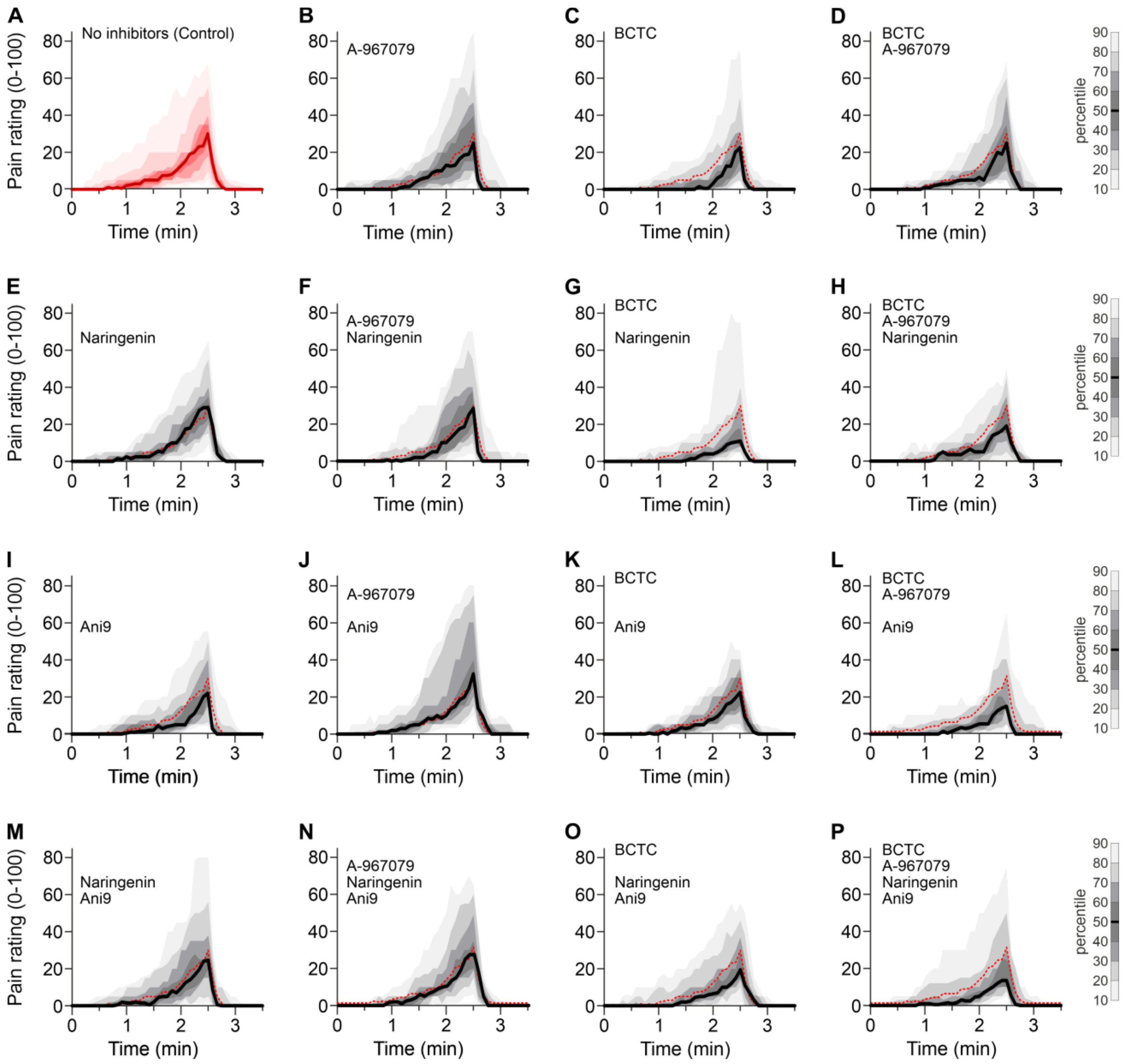

Fig. S3. Pain ratings of visit 2. Each panel shows the distribution of pain ratings over time during increasingly hot control injections up to 52°C in visit 2 obtained from 24 subjects. These pain ratings, plotted against temperature, ader depicted in Figure 2. (**A**) Pain ratings without antagonists. The median of these results serves as a reference in the other panels (dotted red line). (**B-P**) TRPA1 inhibitor A-967079 10 µM is present in panels of columns 2 and 4 and can be compared to the panels left to it. TRPV1 inhibitor BCTC 1 µM is present in columns 3 and 4. Similarly, TRPM3 inhibitor naringenin 20 µM is present in rows 2 and 4 and can be compared to the panels above it. ANO1 inhibitor Ani9 10 µM is present in rows 3 and 4. Note that due to study design all subjects received either control or all four substances together, but not both of these experimental arms (panel A vs P).

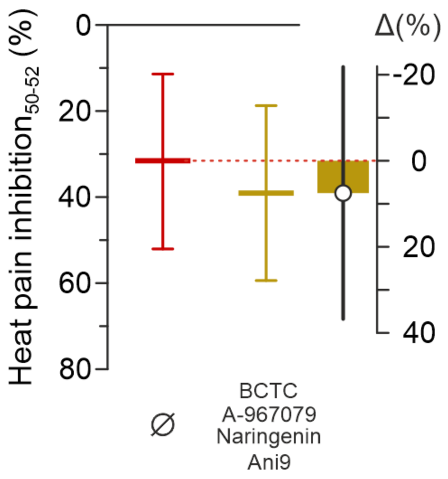

Fig. S4. Heat pain inhibition$_{50\text{-}52}$ in control injections vs. the combination of all four inhibitors of TRPV1, TRPA1, TRPM3 and ANO1. The panel shows the estimated mean ±95% CI for the 24 subjects who had one injection without the substances vs. the other 24 subjects which had one injection with all four substances combined. Right scale indicates the contrast with 95% CI. According to the predefined protocol, this primary endpoint analysis uses HPI$_{50\text{-}52}$ as outcome variable.

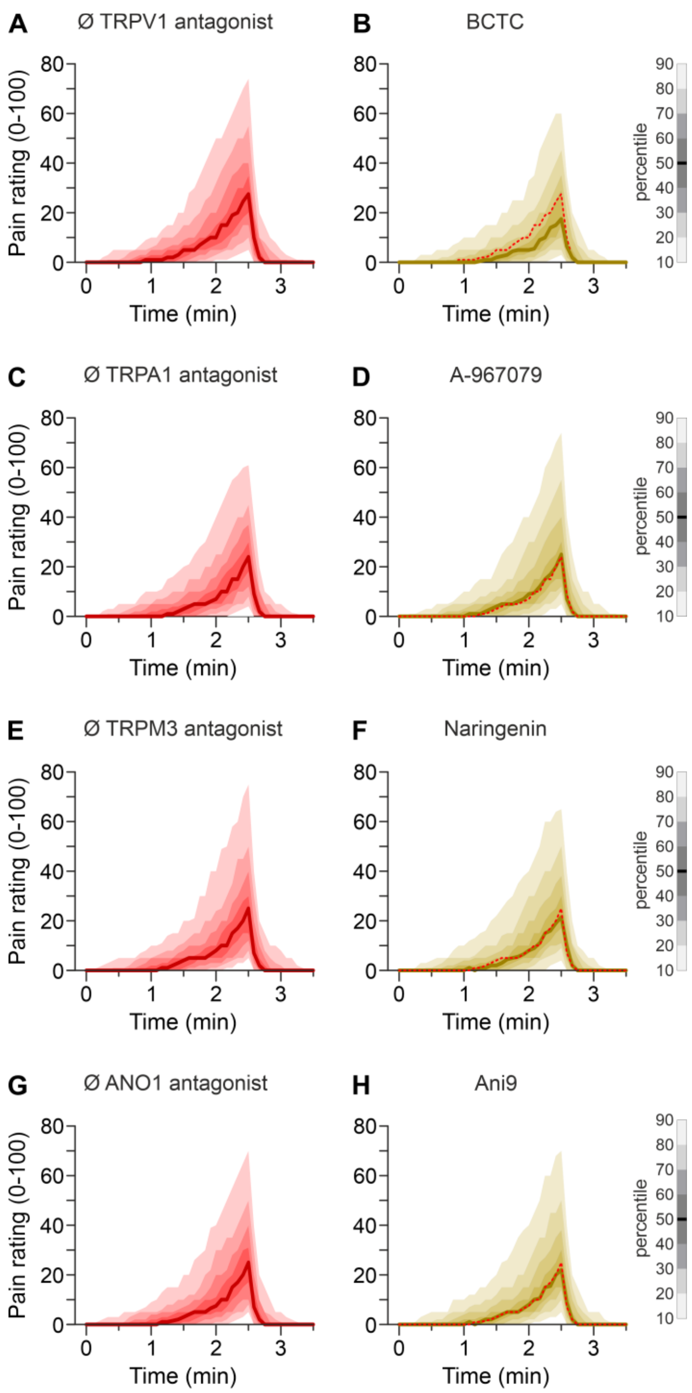

Fig. S5. Heat pain rating time course with and without inhibitors of TRPV1, TRPA1, TRPM3 and ANO1. Each panel shows the distribution of pain ratings over time during increasingly hot control injections up to 52°C in visit 2 obtained from 24 subjects. (**A**) Pain ratings of all injections without and (**B**) with BCTC 1 µM. The effect of BCTC was not different by sex of the subject (P = 0.50). (**C**) Pain ratings of all injections without and (**D**) with A-967079 10 µM. (**E**) Pain ratings of all injections without and (**F**) with naringenin 20 µM. (**G**) Pain ratings of all injections without and (**H**) with Ani9 10 µM.

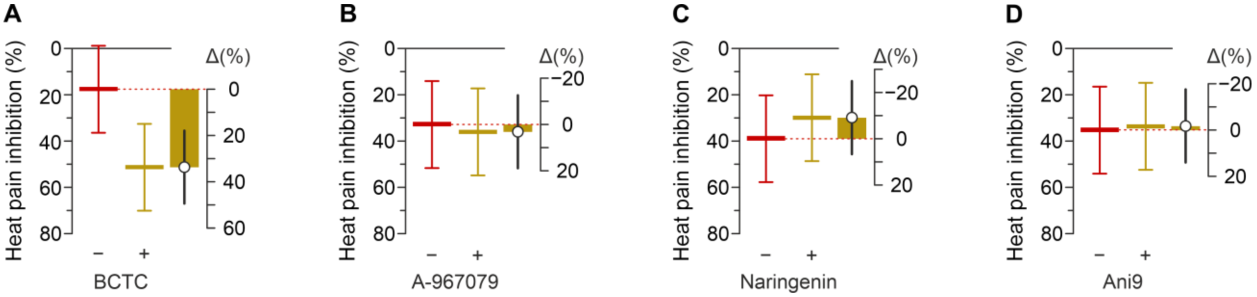

Fig. S6. Heat pain inhibition with and without inhibitors of TRPV1, TRPA1, TRPM3 and ANO1. Each panel shows the estimated mean for all injections with and without the substances. Right scales indicate the contrast with 95% CI. (**A**) For each of the 48 subjects, there were four injections without and four with BCTC 1 µM. When considering all experimental injections administered during visit 2 without using BCTC, including all injections with and without the other inhibitors, there was no evidence for heat inhibition ($p = 0.065$). A habituation to the test situation could explain a tendency towards lower pain ratings at visit 2 without BCTC compared to the hot injections at visit 1. (**B**) Heat pain inhibition was not different for the four injections with A-967079 10 µM, (**C**) naringenin 20 µM and (**D**) Ani9 10 µM compared to the four injections without the respective substance. Per protocol, these main effects analyzed the heat pain inhibition over the full temperature range. Note that half of the injections used to estimate the heat pain inhibition for A-967079, naringenin and Ani9 include BCTC. Therefore, these heat pain inhibition estimates are different from 0.

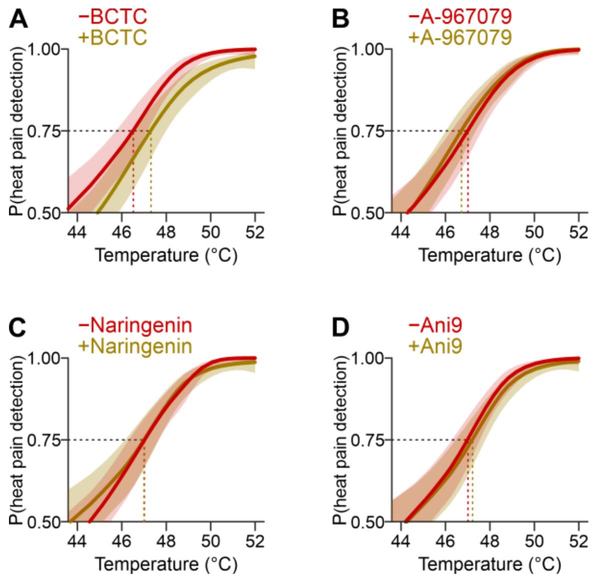

Fig. S7. Modeled probability to distinguish heated and room temperature injections. The model used the individual three injections at room temperature. This was modeled in presence and absence of the respective antagonists. (**A-D**) In addition to Figure 4, the panels additionally visualize the 95% confidence intervals for the injections without the antagonist (light red).

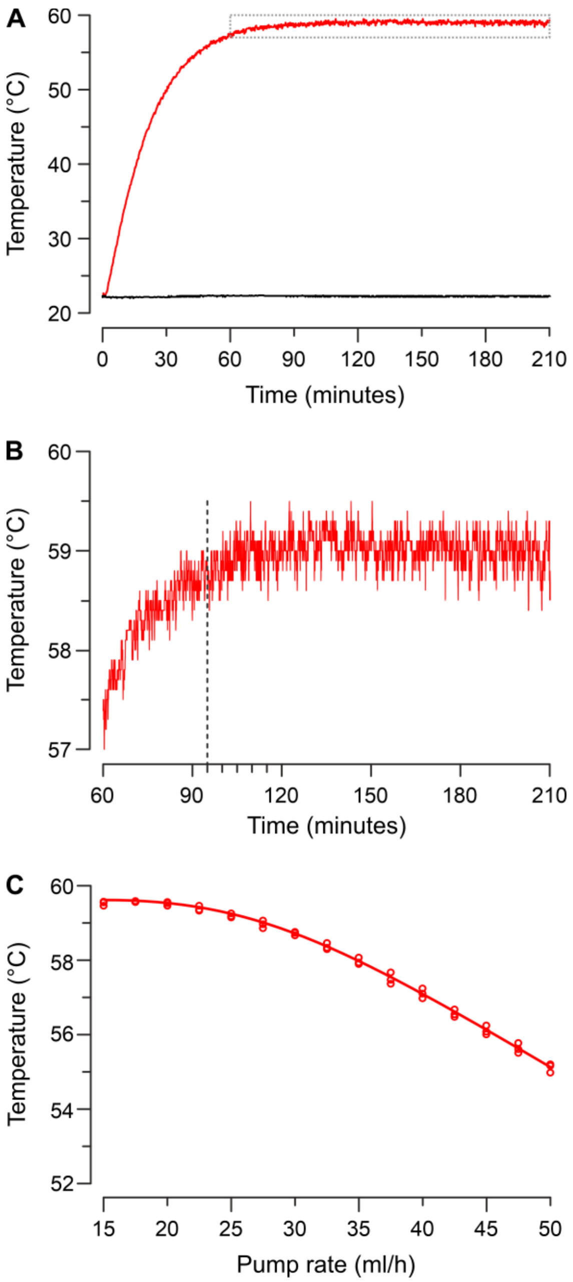

Fig. S8. Heat stimulation by passing fluid over a heat block. (**A**) An aluminum alloy block was fixed on a heating block. The temperature was measured with a thermocouple in the room (black) and at the corner of the aluminum alloy block (red). After activating the heater, set at a constant temperature of 71 °C, it takes about 90 minutes for equilibration of the surface of the aluminum alloy block to 59°C. (**B**) Magnified view of the dashed rectangle from panel a. All experiments started at least 95 minutes after activating the heater, as indicated by the dashed line. (**C**) For the used pump rates of 15–33 ml/h, the temperature at the end of the heat contact remains within a narrow range. At higher pump rates, the contact period would become too short for full thermal equilibration and the heat block contact would need to be increased. Data from three independent measurements were fitted by a quadratic polynomial.

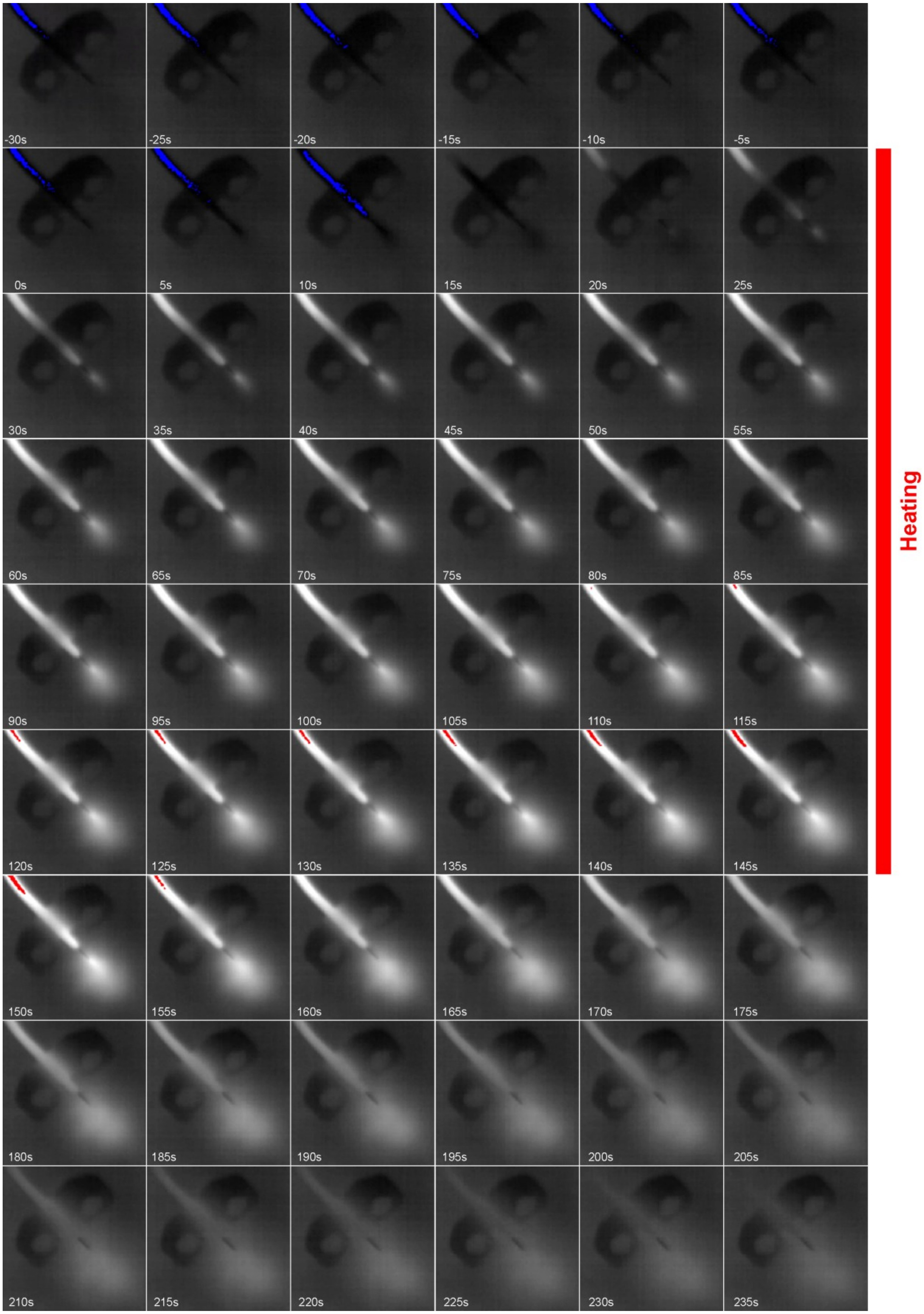
-30s
-25s
-20s
-15s
-10s
-5s
0s
5s
10s
15s
20s
25s
30s
35s
40s
45s
50s
55s
60s
65s
70s
75s
80s
85s
90s
95s
100s
105s
110s
115s
120s
125s
130s
135s
140s
145s
150s
155s
160s
165s
170s
175s
180s
185s
190s
195s
200s
205s
210s
215s
220s
225s
230s
235s
Heating

Figure S9. Skin temperature distribution in the infusion-based heat pain model. From the thermal camera video of a heat stimulation shown in video S1, images were taken every 5 seconds. There area of interest was magnified and contrasted for the montage. The heat 150 s application period is indicated by the horizontal red bar.

**A**	1	2	8	3	7	4	6	5		1	2	8	3	7	4	6	5	**A′**
	−	−	+	+	−	−	+	+	BCTC 1 µM	+	+	−	−	+	+	−	−	
	−	+	+	−	+	−	−	+	A-967079 10 µM	+	−	−	+	−	+	+	−	
	−	+	−	+	−	+	−	+	Naringenin 20 µM	+	−	+	−	+	−	+	−	
	−	+	−	+	+	−	+	−	Ani-9 10 µM	+	−	+	−	−	+	−	+	
**B**	2	3	1	4	8	5	7	6		2	3	1	4	8	5	7	6	**B′**
	−	+	−	−	+	+	−	+	BCTC 1 µM	+	−	+	+	−	−	+	−	
	+	−	−	−	+	+	+	−	A-967079 10 µM	−	+	+	+	−	−	−	+	
	+	+	−	+	−	+	−	−	Naringenin 20 µM	−	−	+	−	+	−	+	+	
	+	+	−	−	−	−	+	+	Ani-9 10 µM	−	−	+	+	+	+	−	−	
**C**	3	4	2	5	1	6	8	7		3	4	2	5	1	6	8	7	**C′**
	+	−	−	+	−	+	+	−	BCTC 1 µM	−	+	+	−	+	−	−	+	
	−	−	+	+	−	−	+	+	A-967079 10 µM	+	+	−	−	+	+	−	−	
	+	+	+	+	−	−	−	−	Naringenin 20 µM	−	−	−	−	+	+	+	+	
	+	−	+	−	−	+	−	+	Ani-9 10 µM	−	+	−	+	+	−	+	−	
**D**	4	5	3	6	2	7	1	8		4	5	3	6	2	7	1	8	**D′**
	−	+	+	+	−	−	−	+	BCTC 1 µM	+	−	−	−	+	+	+	−	
	−	+	−	−	+	+	−	+	A-967079 10 µM	+	−	+	+	−	−	+	−	
	+	+	+	−	+	−	−	−	Naringenin 20 µM	−	−	−	+	−	+	+	+	
	−	−	+	+	+	+	−	−	Ani-9 10 µM	+	+	−	−	−	−	+	+	
**E**	5	6	4	7	3	8	2	1		5	6	4	7	3	8	2	1	**E′**
	+	+	−	−	+	+	−	−	BCTC 1 µM	−	−	+	+	−	−	+	+	
	+	−	−	+	−	+	+	−	A-967079 10 µM	−	+	+	−	+	−	−	+	
	+	−	+	−	+	−	+	−	Naringenin 20 µM	−	+	−	+	−	+	−	+	
	−	+	−	+	+	−	+	−	Ani-9 10 µM	+	−	+	−	−	+	−	+	
**F**	6	7	5	8	4	1	3	2		6	7	5	8	4	1	3	2	**F′**
	+	−	+	+	−	−	+	−	BCTC 1 µM	−	+	−	−	+	+	−	+	
	−	+	+	+	−	−	−	+	A-967079 10 µM	+	−	−	−	+	+	+	−	
	−	−	+	−	+	−	+	+	Naringenin 20 µM	+	+	−	+	−	+	−	−	
	+	+	−	−	−	−	+	+	Ani-9 10 µM	−	−	+	+	+	+	−	−	
**G**	7	8	6	1	5	2	4	3		7	8	6	1	5	2	4	3	**G′**
	−	+	+	−	+	−	−	+	BCTC 1 µM	+	−	−	+	−	+	+	−	
	+	+	−	−	+	+	−	−	A-967079 10 µM	−	−	+	+	−	−	+	+	
	−	−	−	−	+	+	+	+	Naringenin 20 µM	+	+	+	+	−	−	−	−	
	+	−	+	−	−	+	−	+	Ani-9 10 µM	−	+	−	+	+	−	+	−	
**H**	8	1	7	2	6	3	5	4		8	1	7	2	6	3	5	4	**H′**
	+	−	−	−	+	+	+	−	BCTC 1 µM	−	+	+	+	−	−	−	+	
	+	−	+	+	−	−	+	−	A-967079 10 µM	−	+	−	−	+	+	−	+	
	−	−	−	+	−	+	+	+	Naringenin 20 µM	+	+	+	−	+	−	−	−	
	−	−	+	+	+	+	−	−	Ani-9 10 µM	+	+	−	−	−	−	+	+	

Fig. S10. Pre-specified sequences. Sixteen pre-defined sequences for visit 2. Both sequences A-H and sequences A’ to H’ are balanced Williams designs, however sequences A’ to H’ are inverted concerning whether a substance is used or not. Every subject received eight injections in the order 1–8. Within sets of 16 subjects, a sequence was randomly chosen and removed. As a compromise in favor of factorial design, no subject had −/−/−/− and +/+/+/+.

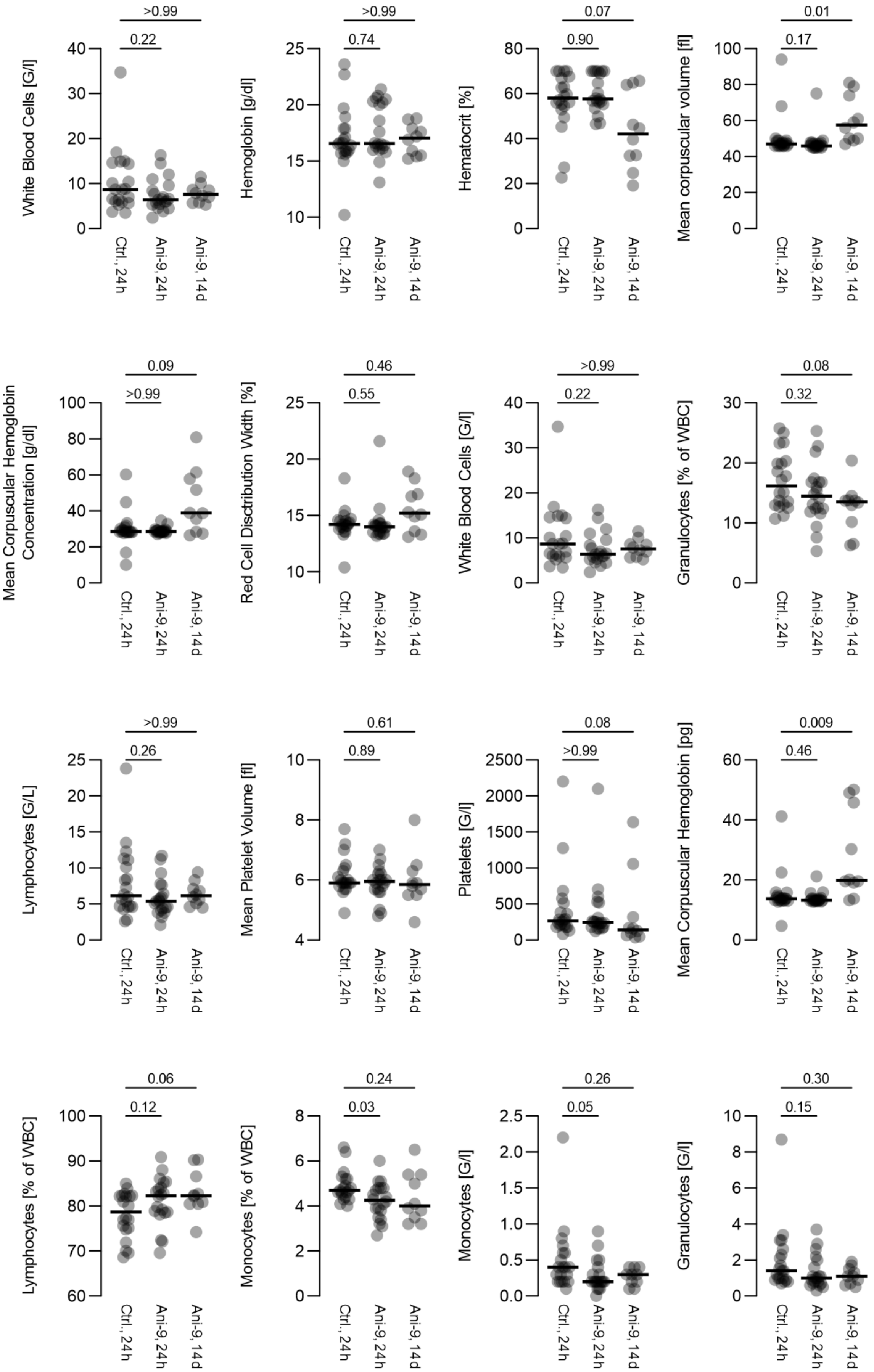
White Blood Cells [G/l]
>0.99
0.22
Hemoglobin [g/dl]
>0.99
0.74
Hematocrit [%]
0.07
0.90
Mean corpuscular volume [fl]
0.01
0.17
Mean Corpuscular Hemoglobin Concentration [g/dl]
0.09
>0.99
Red Cell Discribution Width [%]
0.46
0.55
White Blood Cells [G/l]
>0.99
0.22
Granulocytes [% of WBC]
0.08
0.32
Lymphocytes [G/L]
>0.99
0.26
Mean Platelet Volume [fl]
0.61
0.89
Platelets [G/l]
0.08
>0.99
Mean Corpuscular Hemoglobin [pg]
0.009
0.46
Lymphocytes [% of WBC]
0.06
0.12
Monocytes [% of WBC]
0.24
0.03
Monocytes [G/l]
0.26
0.05
Granulocytes [G/l]
0.30
0.15
Ctrl., 24h
Ani-9, 24h
Ani-9, 14d

Fig. S11. Blood count of Ani9 injected mice. As defined by the EMA ICH guideline M3 (R2), parameters were analyzed after 24 hours and 14 days. There were 20 animals each, either injected with a control solution or Ani9 156 µg/kg (~1000-fold of human dosing, subcutaneous), analyzed after 24 hours. Further, there were 10 animals subcutaneous injected with Ani9 156 µg/kg with analysis after 14 days, showing significant changes in some of the red blood cell indices (Kruskal-Wallis test, Dunn's post-hoc correction).

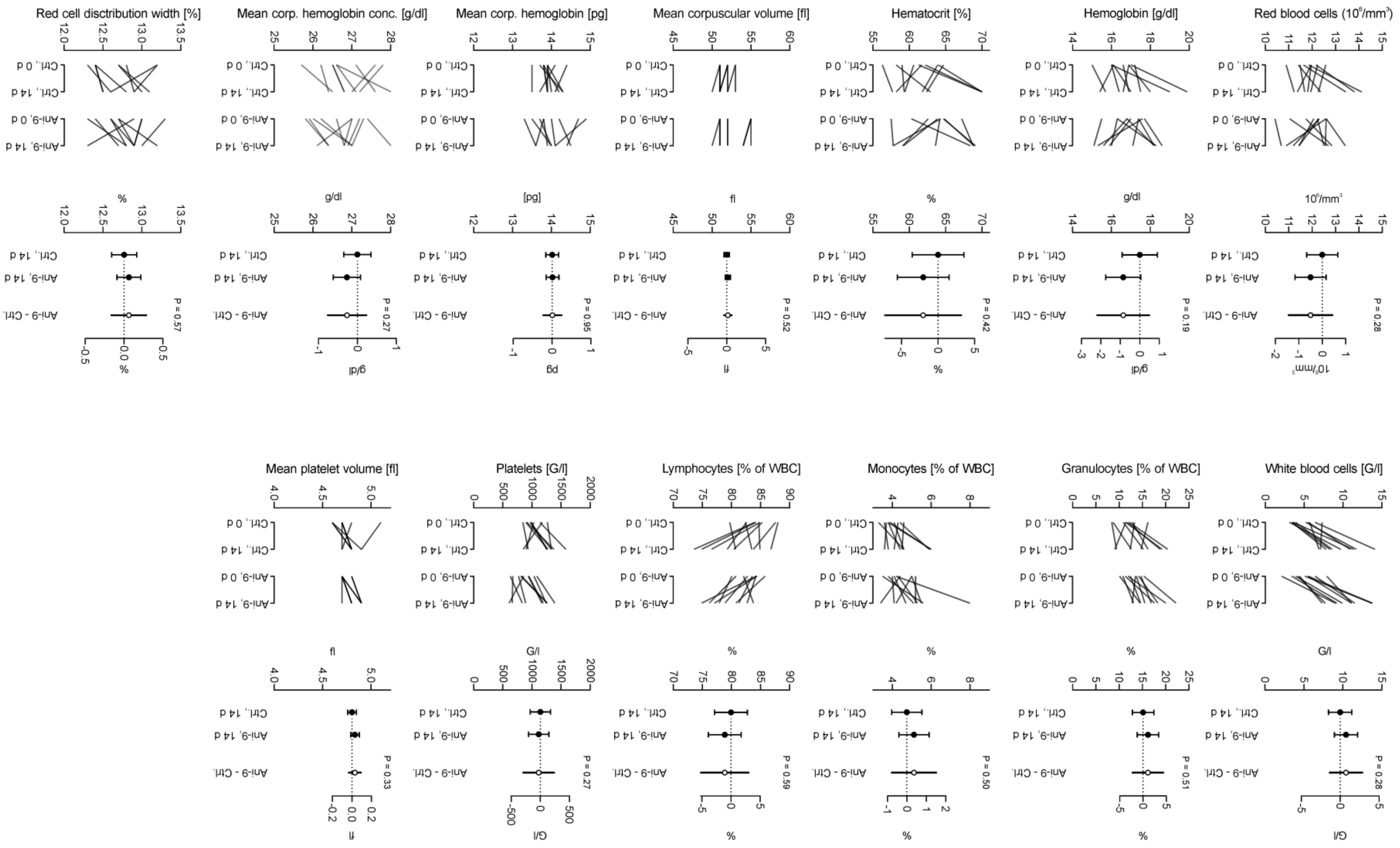

Red cell distribution width [%]
Mean corp. hemoglobin conc. [g/dl]
Mean corp. hemoglobin [pg]
Mean corpuscular volume [fl]
Hematocrit [%]
Hemoglobin [g/dl]
Red blood cells (10^6/mm^3)
P = 0.57
P = 0.27
P = 0.95
P = 0.52
P = 0.42
P = 0.19
P = 0.28
Mean platelet volume [fl]
Platelets [G/l]
Lymphocytes [% of WBC]
Monocytes [% of WBC]
Granulocytes [% of WBC]
White blood cells [G/l]
P = 0.33
P = 0.27
P = 0.59
P = 0.50
P = 0.51
P = 0.28
Ctrl., 0 d
Ctrl., 14 d
Ani-9, 0 d
Ani-9, 14 d
Ani-9 - Ctrl.

Fig. S12. Blood count of Ani9 injected mice. This was determined in an independent experiment following the experiment presented in extended data figure 9, as some values were different for Ani9 14 days after the injection. This required investigation with 14 d control animals, which are not part of the EMA ICH guideline M3 (R2). Spaghetti plots were chosen due to repeated measurements in the same animals. Filled symbols represent the least squares mean of the indicated group after 14 d, corrected for interindividual differences at 0 d. Open circles represent the estimated difference between groups at 14 d. Error bars are 95% confidence intervals. Addition of these controls again showed a time-dependent change in some parameters, but independent of the Ani9 treatment.

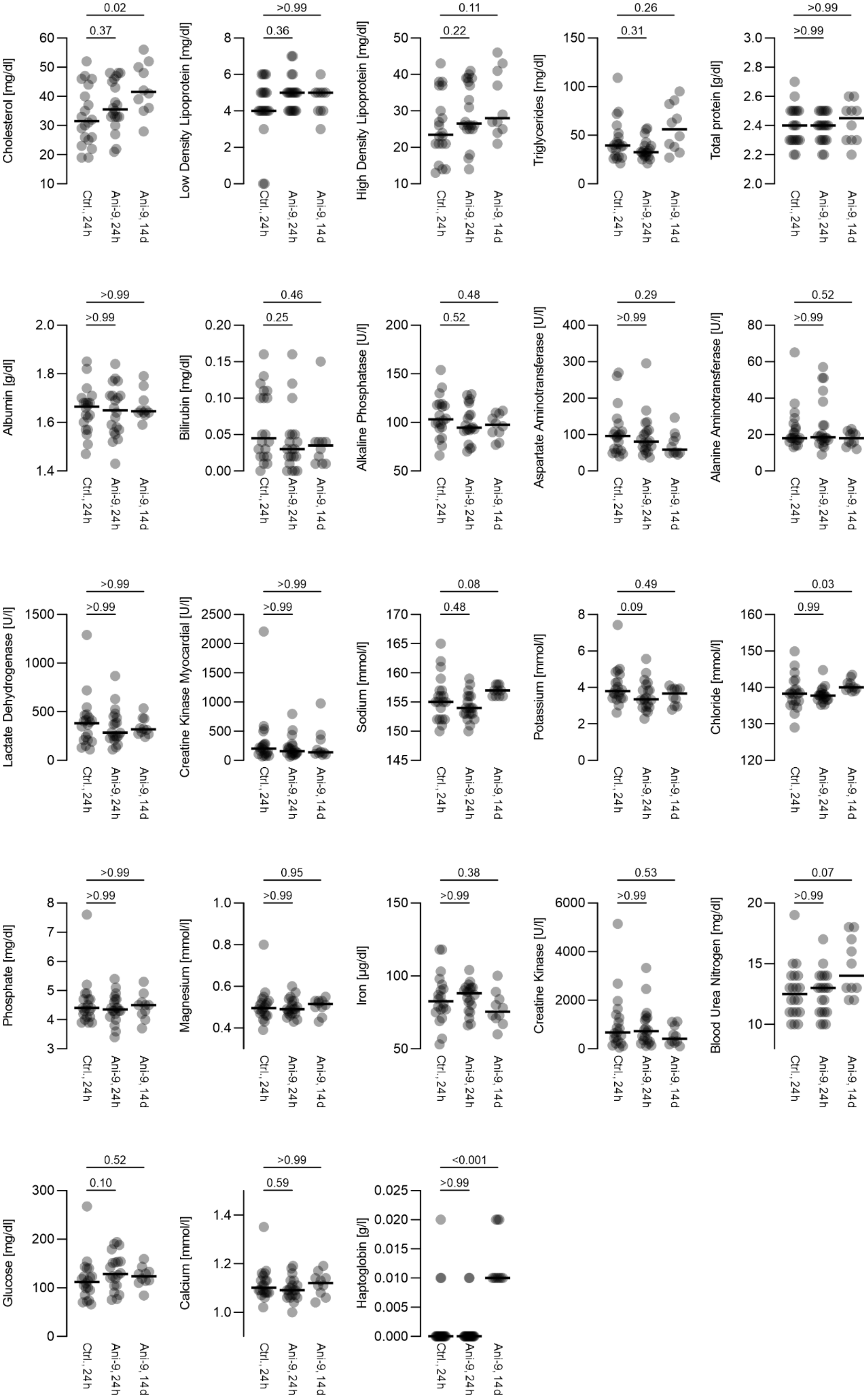
Cholesterol [mg/dl]
0.02
0.37
Low Density Lipoprotein [mg/dl]
>0.99
0.36
High Density Lipoprotein [mg/dl]
0.11
0.22
Triglycerides [mg/dl]
0.26
0.31
Total protein [g/dl]
>0.99
>0.99
Albumin [g/dl]
>0.99
>0.99
Bilirubin [mg/dl]
0.46
0.25
Alkaline Phosphatase [U/l]
0.48
0.52
Aspartate Aminotransferase [U/l]
0.29
>0.99
Alanine Aminotransferase [U/l]
0.52
>0.99
Lactate Dehydrogenase [U/l]
>0.99
>0.99
Creatine Kinase Myocardial [U/l]
>0.99
>0.99
Sodium [mmol/l]
0.08
0.48
Potassium [mmol/l]
0.49
0.09
Chloride [mmol/l]
0.03
0.99
Phosphate [mg/dl]
>0.99
>0.99
Magnesium [mmol/l]
0.95
>0.99
Iron [µg/dl]
0.38
>0.99
Creatine Kinase [U/l]
0.53
>0.99
Blood Urea Nitrogen [mg/dl]
0.07
>0.99
Glucose [mg/dl]
0.52
0.10
Calcium [mmol/l]
>0.99
0.59
Haptoglobin [g/l]
<0.001
>0.99
Ctrl., 24h
Ani-9, 24h
Ani-9, 14d

Fig. S13. Clinical chemistry of Ani9 injected mice. As defined by the EMA ICH guideline M3 (R2), parameters were analyzed after 24 hours and 14 days. There were 20 animals each, either injected with a control solution or Ani9 156 µg/kg (~1000-fold of human dosing, subcutaneous), analyzed after 24 hours. Further, there were 10 animals subcutaneous injected with Ani9 156 µg/kg with analysis after 14 days. No relevant changes were observed (Kruskal-Wallis test, Dunn's post-hoc correction).

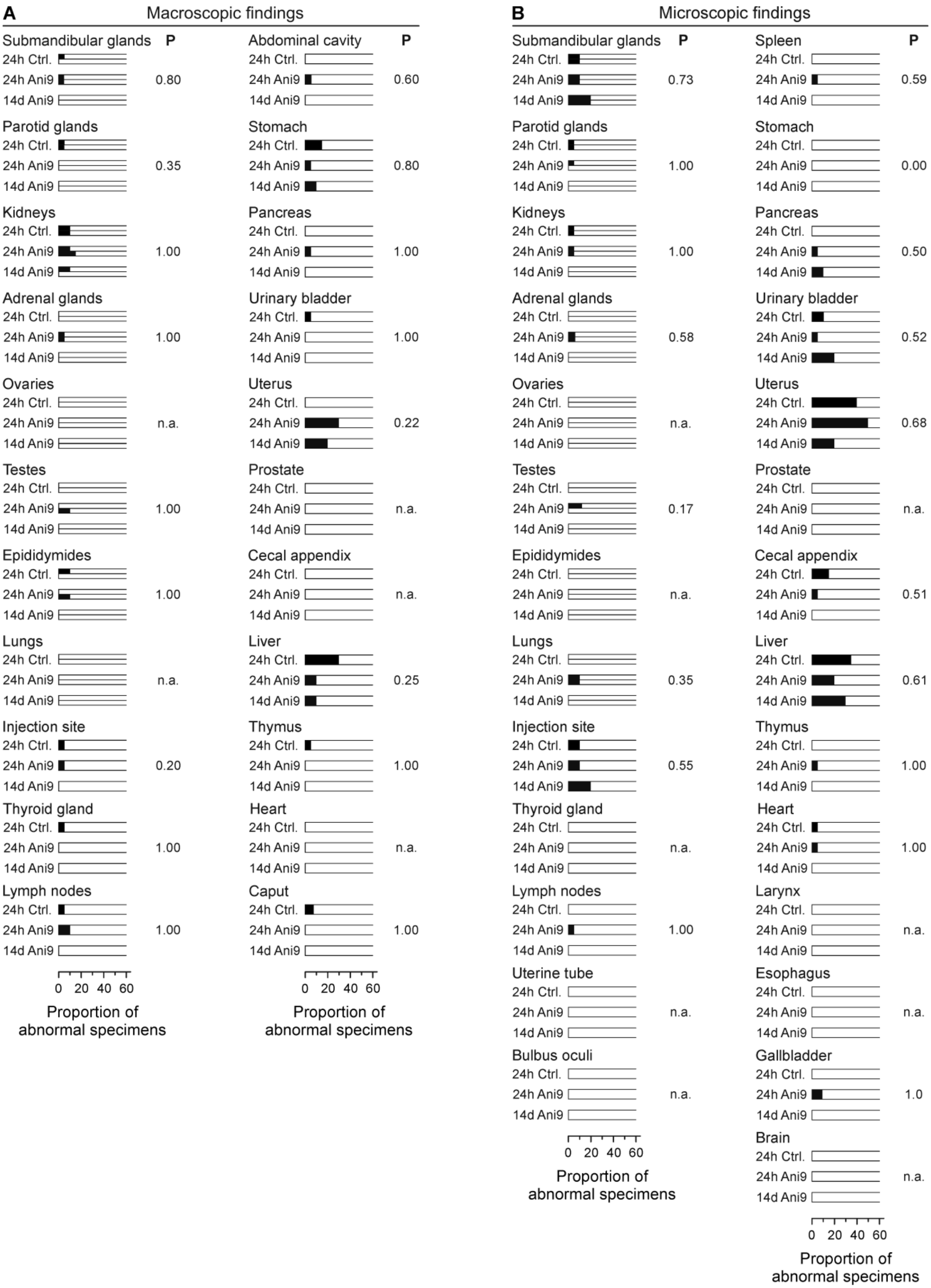
A
Macroscopic findings
Submandibular glands
P
24h Ctrl.
24h Ani9
0.80
14d Ani9
Parotid glands
0.35
Kidneys
1.00
Adrenal glands
1.00
Ovaries
n.a.
Testes
1.00
Epididymides
1.00
Lungs
n.a.
Injection site
0.20
Thyroid gland
1.00
Lymph nodes
1.00
Abdominal cavity
0.60
Stomach
0.80
Pancreas
1.00
Urinary bladder
1.00
Uterus
0.22
Prostate
n.a.
Cecal appendix
n.a.
Liver
0.25
Thymus
1.00
Heart
n.a.
Caput
1.00
0 20 40 60
Proportion of abnormal specimens
B
Microscopic findings
Submandibular glands
0.73
Parotid glands
1.00
Kidneys
1.00
Adrenal glands
0.58
Ovaries
n.a.
Testes
0.17
Epididymides
n.a.
Lungs
0.35
Injection site
0.55
Thyroid gland
n.a.
Lymph nodes
1.00
Uterine tube
n.a.
Bulbus oculi
n.a.
Spleen
0.59
Stomach
0.00
Pancreas
0.50
Urinary bladder
0.52
Uterus
0.68
Prostate
n.a.
Cecal appendix
0.51
Liver
0.61
Thymus
1.00
Heart
1.00
Larynx
n.a.
Esophagus
n.a.
Gallbladder
1.0
Brain
n.a.
0 20 40 60
Proportion of abnormal specimens

Fig. S14. Macroscopy and histopathology of Ani9 injected mice. (**A**) Macroscopic and (**B**) histopathologic results. Each subpanel shows the proportion of abnormal specimens within each group as horizontal bars. For paired organs, two bars are shown. Next to each bar, the plotted proportion is given as a number. The P-value refers to the hypothesis that the proportion of abnormal specimens differs between the three groups. In case there was no pathology in all groups, no P-value can be calculated (not applicable n.a.).

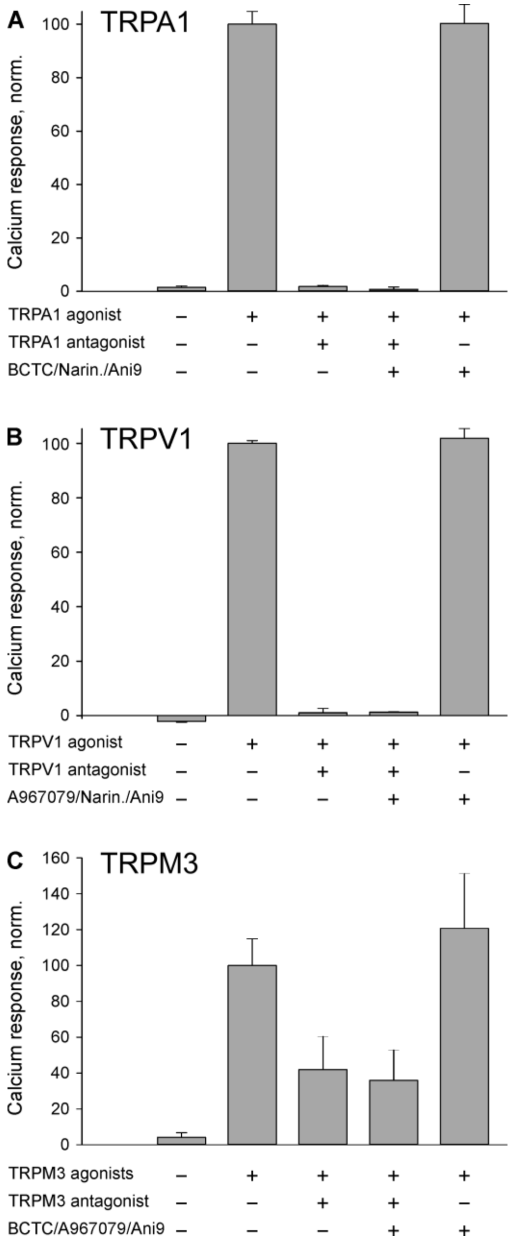

Fig. S15. Antagonist specificity regarding human TRPA1, TRPV1 and TRPM3. (**A**) HEK293t cells expressing human TRPA1 did not respond to solvent controls, but to TRPA1 agonist JT010 0.1 µM. This was fully inhibited by A-967079 2 µM. Further addition of BCTC 0.2 µM, naringenin 50 µM and Ani9 10 µM did not alter the inhibition by A-967079, nor did the other three antagonists alter the response to AITC. (**B**) Similar capsaicin 0.2 µM activation of expressed human TRPV1 was fully inhibited by BCTC 0.2 µM, this inhibition was not different in the presence of the other three agonists, and the latter did not affect the response to capsaicin. (**C**) CIM0216 2.1 µM and pregnenolone sulfate 2.1 µM were combined to activate human TRPM3. As previously reported, this generates a supra-additive effect, which could be partially inhibited by the naringenin 50 µM. Again, this inhibition was not different in the presence of the other three agonists, and the latter did not significantly affect the response to the agonist combination. Data are mean + SD of at least three independent measurements.

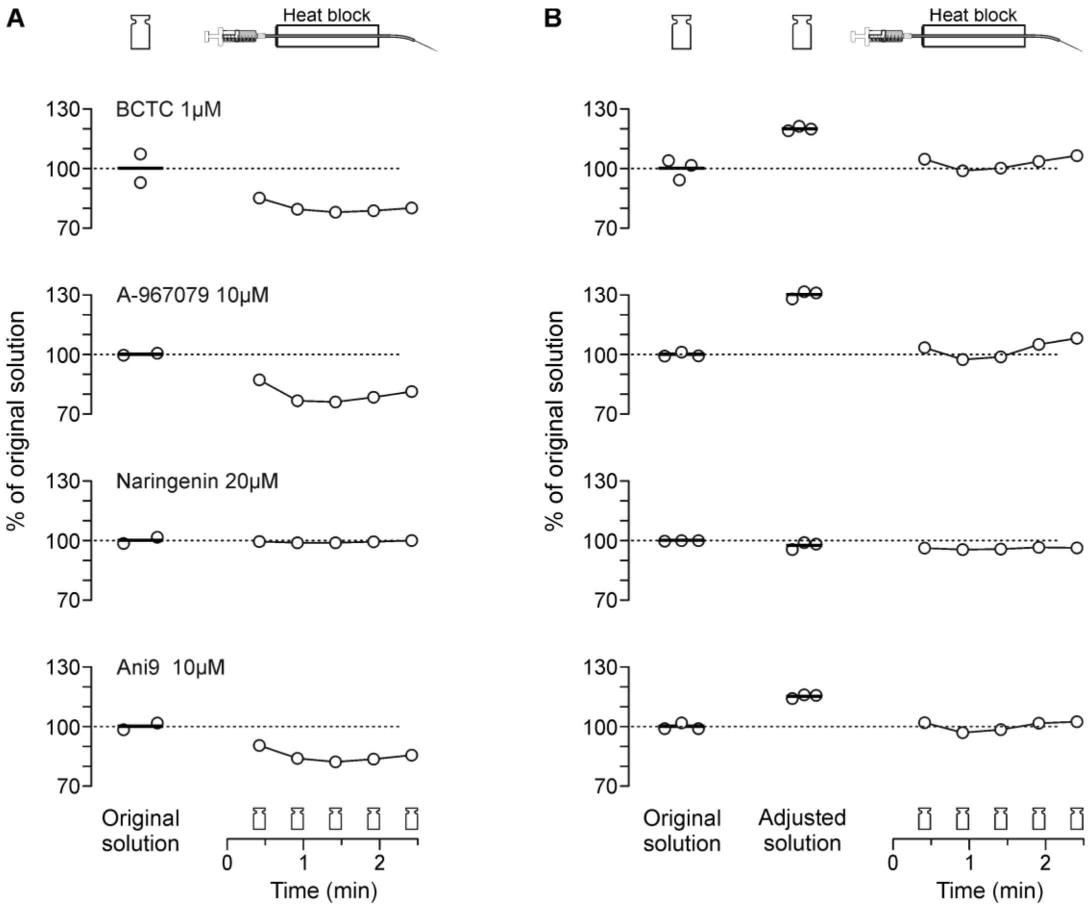

Fig. S16. Quantification of injected antagonists. (**A**) High-performance liquid chromatography was used to quantify the substances from the outlet of the setup used for the human subjects. Substance losses might occur due to adsorption or lack of heat stability. First, pre-specified concentrations were generated and handled identical to the psychophysical experiments, including contact to syringe and tubing, and being heated before collection of the solution from the needle tip. The absence of a unidirectional trend in the heated measurements argues in favor of heat stability. (**B**) Based on the results, the concentrations were adjusted, and in a subsequent measurement, they were within narrow margins of the target concentrations.

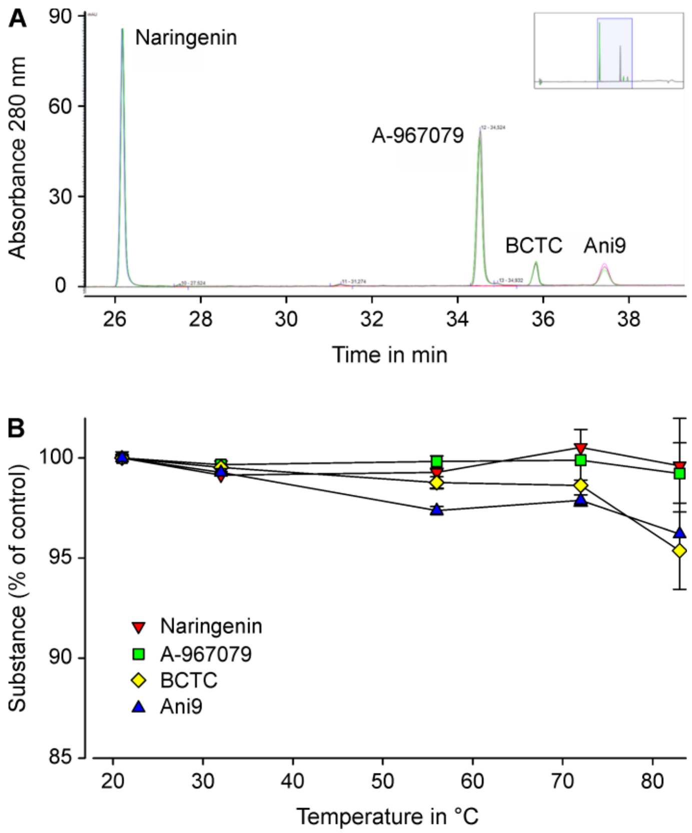

Fig. S17. Thermal stability of antagonists by high-performance liquid chromatography. (**A**) The absorbance at 280 nm of the antagonists was determined in four independent runs. The four antagonists were mixed and analyzed (light blue), or after exposure to 72°C for 10 min (black). The respective absorption peaks were similar to the separate runs with only one substance; Naringenin (red), A-967079 (green), BCTC (yellow), Ani9 (blue). No additional peaks, indicating adducts or fission products, were detected, indicating that there is no chemical change due to compounding of the antagonists. (**B**) Recovery of the combined antagonists after 3 minutes of heat exposure for 3 minutes.

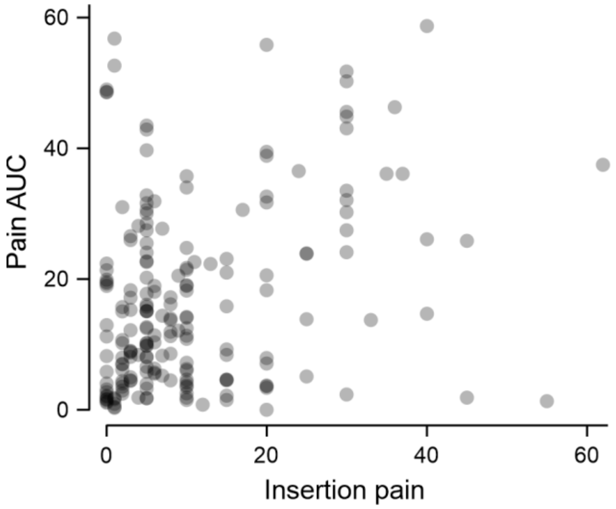

Fig. S18. Relationship between insertion pain and of heat-induced pain. There is a positive correlation (Spearman $\rho = 0.322$, $p < 0.001$) between cannula insertion pain and heat-induced pain. As both cannula insertion pain and heat-induced pain might be associated with local nerve fiber density, a positive correlation was expected and therefore the insertion pain considered as covariate in the preplanned analysis. The result supports the consideration of insertion pain also in future studies.

Ethics vote for N = 32 + 4

↓

Completion of internal pilot N = 16

↓

Simulations based on variances and covariances of internal pilot

↓

Sample size:

	N = 32	N = 48
Primary endpoint:	68.7 %	85.7 %
3-way interaction:	17.3 %	24.5 %
2-way interaction:	51.0 %	65.0 %
Main effects:	97.0 %	> 99.0 %

↓

Amendment of ethics vote for N = 48 + 4

↓

51 included subjects

↓

51 subjects completed visit 1

↓

48 subjects completed visit 2

Fig. S19. Study flow chart. The study was planned for an internal pilot after 16 subjects, as multiple of 16 represent completion of Williams squares for sequence permutation. To allow extension to 32 subjects, the latter had been pre-approved with four subjects surplus accounting for a dropout rate of 10%. The simulations based on the internal pilot showed that with N = 32 the study was underpowered for the primary hypothesis as well as for interaction terms. Therefore, the study protocol was amended for 48 complete subjects to have >80% power for the primary endpoint. In total, 51 subjects were included, as 2 did not attend the second visit due to time limitations, and in one subject loss of a (blinded) syringe did not allow the completion of the protocol.

Inclusion criteria	Age between 18 and 70 years
	Full legal capacity
Exclusion criteria	Participant of another study, ongoing or within the last 4 weeks
	Medication intake (except contraception) or drug abuse
	Female subjects: Positive pregnancy test or breastfeeding
	Body temperature above 38°C, diagnostically verified
	Known allergic diseases, in particular asthmatic disorders and skin diseases,<br>known allergic reactions to citrus fruits
	Sensory deficit, skin disease<br>or hematoma of unknown origin in physical examination of the test site
	Symptoms of a respiratory tract infection (Covid-19 related criterion)

Table S1. Inclusion and exclusion criteria

Effect	Numerator degrees of freedom	Denominator degrees of freedom	F	p
Period	7	265	0.95	0.47
log(cannula_insertion_pain)	1	280	1.65	0.20
TRPV1_antagonist	1	283	17.46	<.0001
TRPA1_antagonist	1	283	0.16	0.69
TRPM3_antagonist	1	283	1.26	0.26
ANO1_antagonist	1	285	0.04	0.84
TRPV1_antagonist*TRPA1_antagonist	1	285	0.02	0.89
TRPV1_antagonist*TRPM3_antagonist	1	285	0.00	0.96
TRPV1_antagonist*ANO1_antagonist	1	283	0.93	0.35
TRPA1_antagonist*TRPM3_antagonist	1	285	0.03	0.86
TRPA1_antagonist*ANO1_antagonist	1	283	0.04	0.84
TRPM3_antagonist*ANO1_antagonist	1	284	0.62	0.43
TRPV1_antagonist*TRPA1_antagonist*TRPM3_antagonist	1	283	2.00	0.16
TRPV1_antagonist*TRPA1_antagonist*ANO1_antagonist	1	45.9	0.56	0.46
TRPV1_antagonist*TRPM3_antagonist*ANO1_antagonist	1	285	0.13	0.72
TRPA1_antagonist*TRPM3_antagonist*ANO1_antagonist	1	285	0.13	0.72

Table S2. Type 3 tests of fixed effects: Heat pain inhibition. Table provides for main effects, two-way and three-way interactions, the degrees of freedom in the numerator and denominator, as well as the respective F and p values.

Video S1. Time course of the skin surface temperature. The scale bar at the left indicates how the 256 gray levels correspond to the temperature range 30–48°C. Pixels below 30°C temperature above are blue, pixels above 48°C red, the latter includes the heat block. The video is 9 seconds long has been sped up 30 times from the original 270 seconds and is otherwise unaltered. Heat stimulation occurs in the accelerated video from second 2-6, the increasing pump speed as well as the analog can be heard in the audio channel. The winged infusion set serves as a scaling reference. Images of the area of interest every 5 s are depicted in figure S9.

REFERENCES AND NOTES

1. D. Julius, TRP channels and pain. *Annu. Rev. Cell Dev. Biol.* **29**, 355–384 (2013).

2. C. Peirs, R. P. Seal, Neural circuits for pain: Recent advances and current views. *Science* **354**, 578–584 (2016).

3. J. Vriens, B. Nilius, T. Voets, Peripheral thermosensation in mammals. *Nat. Rev. Neurosci.* **15**, 573–589 (2014).

4. M. J. Caterina, A. Leffler, A. B. Malmberg, W. J. Martin, J. Trafton, K. R. Petersen-Zeitz, M. Koltzenburg, A. I. Basbaum, D. Julius, Impaired nociception and pain sensation in mice lacking the capsaicin receptor. *Science* **288**, 306–313 (2000).

5. J. Vriens, G. Owsianik, T. Hofmann, S. E. Philipp, J. Stab, X. Chen, M. Benoit, F. Xue, A. Janssens, S. Kerselaers, J. Oberwinkler, R. Vennekens, T. Gudermann, B. Nilius, T. Voets, TRPM3 is a nociceptor channel involved in the detection of noxious heat. *Neuron* **70**, 482–494 (2011).

6. D. M. Bautista, S. E. Jordt, T. Nikai, P. R. Tsuruda, A. J. Read, J. Poblete, E. N. Yamoah, A. I. Basbaum, D. Julius, TRPA1 mediates the inflammatory actions of environmental irritants and proalgesic agents. *Cell* **124**, 1269–1282 (2006).

7. C. J. Woodbury, M. Zwick, S. Wang, J. J. Lawson, M. J. Caterina, M. Koltzenburg, K. M. Albers, H. R. Koerber, B. M. Davis, Nociceptors lacking TRPV1 and TRPV2 have normal heat responses. *J. Neurosci.* **24**, 6410–6415 (2004).

8. S. M. Huang, X. Li, Y. Yu, J. Wang, M. J. Caterina, TRPV3 and TRPV4 ion channels are not major contributors to mouse heat sensation. *Mol. Pain* **7**, 37 (2011).

9. I. Vandewauw, K. De Clercq, M. Mulier, K. Held, S. Pinto, N. Van Ranst, A. Segal, T. Voet, R. Vennekens, K. Zimmermann, J. Vriens, T. Voets, A TRP channel trio mediates acute noxious heat sensing. *Nature* **555**, 662–666 (2018).

10. H. Cho, Y. D. Yang, J. Lee, B. Lee, T. Kim, Y. Jang, S. K. Back, H. S. Na, B. D. Harfe, F. Wang, R. Raouf, J. N. Wood, U. Oh, The calcium-activated chloride channel anoctamin 1 acts as a heat sensor in nociceptive neurons. *Nat. Neurosci.* **15**, 1015–1021 (2012).

11. S. Heber, M. Gold-Binder, C. I. Ciotu, M. Witek, N. Ninidze, H.-G. Kress, M. J. M. Fischer, A human TRPA1-specific pain model. *J. Neurosci.* **39**, 3845–3855 (2019).

12. S. Heber, C. I. Ciotu, G. Hartner, M. Gold-Binder, N. Ninidze, A. Gleiss, H.-G. Kress, M. J. M. Fischer, TRPV1 antagonist BCTC inhibits pH 6.0-induced pain in human skin. *Pain* **161**, 1532–1541 (2020).

13. I. Straub, F. Mohr, J. Stab, M. Konrad, S. E. Philipp, J. Oberwinkler, M. Schaefer, Citrus fruit and fabacea secondary metabolites potently and selectively block TRPM3. *Br. J. Pharmacol.* **168**, 1835–1850 (2013).

14. Y. Seo, H. K. Lee, J. Park, D.-K. Jeon, S. Jo, M. Jo, W. Namkung, Ani9, a novel potent small-molecule ANO1 inhibitor with negligible effect on ANO2. *PLOS ONE* **11**, e0155771 (2016).

15. J. Wang, S.-W. Ou, Y.-J. Wang, Distribution and function of voltage-gated sodium channels in the nervous system. *Channels* **11**, 534–554 (2017).

16. D. L. Bennett, A. J. Clark, J. Huang, S. G. Waxman, S. D. Dib-Hajj, The role of voltage-gated sodium channels in pain signaling. *Physiol. Rev.* **99**, 1079–1151 (2019).

17. L. Cao, A. McDonnell, A. Nitzsche, A. Alexandrou, P.-P. Saintot, A. J. C. Loucif, A. R. Brown, G. Young, M. Mis, A. Randall, S. G. Waxman, P. Stanley, S. Kirby, S. Tarabar, A. Gutteridge, R. Butt, R. M. McKernan, P. Whiting, Z. Ali, J. Bilsland, E. B. Stevens, Pharmacological reversal of a pain phenotype in iPSC-derived sensory neurons and patients with inherited erythromelalgia. *Sci. Transl. Med.* **8**, 335ra56 (2016).

18. M. Eberhardt, T. Stueber, J. de la Roche, C. Herzog, A. Leffler, P. W. Reeh, K. Kistner, TRPA1 and TRPV1 are required for lidocaine-evoked calcium influx and neuropeptide release but not cytotoxicity in mouse sensory neurons. *PLOS ONE* **12**, e0188008 (2017).

19. P. Chevrier, K. Vijayaragavan, M. Chahine, Differential modulation of Nav1.7 and Nav1.8 peripheral nerve sodium channels by the local anesthetic lidocaine. *Br. J. Pharmacol.* **142**, 576–584 (2004).

20. D. C. Rosenberger, U. Binzen, R.-D. Treede, W. Greffrath, The capsaicin receptor TRPV1 is the first line defense protecting from acute non damaging heat: A translational approach. *J. Transl. Med.* **18**, 28 (2020).

21. P. Manitpisitkul, A. Mayorga, K. Shalayda, M. De Meulder, G. Romano, C. Jun, J. A. Moyer, Safety, tolerability and pharmacokinetic and pharmacodynamic learnings from a double-blind, randomized, placebo-controlled, sequential group first-in-human study of the TRPV1 antagonist, JNJ-38893777, in healthy men. *Clin. Drug Investig.* **35**, 353–363 (2015).

22. M. C. Rowbotham, W. Nothaft, R. W. Duan, Y. Wang, C. Faltynek, S. McGaraughty, K. L. Chu, P. Svensson, Oral and cutaneous thermosensory profile of selective TRPV1 inhibition by ABT-102 in a randomized healthy volunteer trial. *Pain* **152**, 1192–1200 (2011).

23. R. Rolke, R. Baron, C. Maier, T. R. Tölle, D. R. Treede, A. Beyer, A. Binder, N. Birbaumer, F. Birklein, I. C. Bötefür, S. Braune, H. Flor, V. Huge, R. Klug, G. B. Landwehrmeyer, W. Magerl, C. Maihöfner, C. Rolko, C. Schaub, A. Scherens, T. Sprenger, M. Valet, B. Wasserka, Quantitative sensory testing in the German Research Network on Neuropathic Pain (DFNS): Standardized protocol and reference values. *Pain* **123**, 231–243 (2006).

24. T. Hoffmann, S. K. Sauer, R. E. Horch, P. W. Reeh, Projected pain from noxious heat stimulation of an exposed peripheral nerve—A case report. *Eur. J. Pain* **13**, 35–37 (2009).

25. Y. Takayama, D. Uta, H. Furue, M. Tominaga, Pain-enhancing mechanism through interaction between TRPV1 and anoctamin 1 in sensory neurons. *Proc. Natl. Acad. Sci. U.S.A.* **112**, 5213–5218 (2015).

26. S. Shah, C. M. Carver, P. Mullen, S. Milne, V. Lukacs, M. S. Shapiro, N. Gamper, Local Ca^{2+} signals couple activation of TRPV1 and ANO1 sensory ion channels. *Sci. Signal.* **13**, eaaw7963 (2020).

27. K. J. Valenzano, E. R. Grant, G. Wu, M. Hachicha, L. Schmid, L. Tafesse, Q. Sun, Y. Rotshteyn, J. Francis, J. Limberis, S. Malik, E. R. Whittemore, D. Hodges, N-(4-tertiarybutylphenyl)-4-(3-

chloropyridin-2-yl)tetrahydropyrazine −1(2H)-carbox-amide (BCTC), a novel, orally effective vanilloid receptor 1 antagonist with analgesic properties: I. In vitro characterization and pharmacokinetic properties. *J. Pharmacol. Exp. Ther.* **306**, 377–386 (2003).

28. M. G. Schwarz, B. Namer, P. W. Reeh, M. J. M. Fischer, TRPA1 and TRPV1 antagonists do not inhibit human acidosis-induced pain. *J. Pain* **18**, 526–534 (2017).

29. H.-J. Behrendt, T. Germann, C. Gillen, H. Hatt, R. Jostock, Characterization of the mouse cold-menthol receptor TRPM8 and vanilloid receptor type-1 VR1 using a fluorometric imaging plate reader (FLIPR) assay. *Br. J. Pharmacol.* **141**, 737–745 (2004).

30. D. Reubish, D. Emerling, J. Defalco, D. Steiger, C. Victoria, F. Vincent, Functional assessment of temperature-gated ion-channel activity using a real-time PCR machine. *Biotechniques* **47**, iii–ix (2009).

31. B. Vilar, C.-H. Tan, P. A. McNaughton, Heat detection by the TRPM2 ion channel. *Nature* **584**, E5–E12 (2020).

32. C.-H. Tan, P. A. McNaughton, The TRPM2 ion channel is required for sensitivity to warmth. *Nature* **536**, 460–463 (2016).

33. K. Song, H. Wang, G. B. Kamm, J. Pohle, F. C. Reis, P. Heppenstall, H. Wende, J. Siemens, The TRPM2 channel is a hypothalamic heat sensor that limits fever and can drive hypothermia. *Science* **353**, 1393–1398 (2016).

34. A. H. Bretag, Synthetic interstitial fluid for isolated mammalian tissue. *Life Sci.* **8**, 319–329 (1969).

35. A. R. Moritz, F. C. Henriques, Studies of thermal injury: II. The relative importance of time and surface temperature in the causation of cutaneous burns. *Am. J. Pathol.* **23**, 695–720 (1947).

36. K. Hinkelmann, O. Kempthorne, *Design and Analysis of Experiments: Volume 2: Advanced Experimental Design* (Wiley-Interscience, ed. 1, 2005).

37. J. Renugadevi, S. M. Prabu, Cadmium-induced hepatotoxicity in rats and the protective effect of naringenin. *Exp. Toxicol. Patho.* **62**, 171–181 (2010).

38. M. Shulman, M. Cohen, A. Soto-Gutierrez, H. Yagi, H. Wang, J. Goldwasser, C. W. Lee-Parsons, O. Benny-Ratsaby, M. L. Yarmush, Y. Nahmias, Enhancement of naringenin bioavailability by complexation with hydroxypropoyl-β-cyclodextrin. [corrected]. *PLOS ONE* **6**, e18033 (2011).

39. S. Wanwimolruk, P. V. Marquez, Variations in content of active ingredients causing drug interactions in grapefruit juice products sold in California. *Drug Metabol. Drug Interact.* **21**, 233–243 (2006).

40. J. Vriens, K. Held, A. Janssens, B. I. Tóth, S. Kerselaers, B. Nilius, R. Vennekens, T. Voets, Opening of an alternative ion permeation pathway in a nociceptor TRP channel. *Nat. Chem. Biol.* **10**, 188–195 (2014).

41. T. Cheng, Y. Zhao, X. Li, F. Lin, Y. Xu, X. Zhang, Y. Li, R. Wang, L. Lai, Computation of octanol-water partition coefficients by guiding an additive model with knowledge. *J. Chem. Inf. Model.* **47**, 2140–2148 (2007).

42. D. Chapman, C. Benedict, H. B. Schiöth, Experimenter gender and replicability in science. *Sci*. *Adv*. **4**, 10.1126/sciadv.1701427.37 (2018).

43. O. A Alabas, O. A. Tashani, G. Tabasam, M. I. Johnson, Gender role affects experimental pain responses: A systematic review with meta-analysis. *Eur*. *J*. *Pain Lond*. *Engl*. **16**, 1211–1223 (2012).
